# Heme oxygenase-1 modulates ferroptosis by fine-tuning levels of intracellular iron and reactive oxygen species of macrophages in response to *Bacillus Calmette-Guerin* infection

**DOI:** 10.3389/fcimb.2022.1004148

**Published:** 2022-09-23

**Authors:** Chenjie Ma, Xiaoling Wu, Xu Zhang, Xiaoming Liu, Guangcun Deng

**Affiliations:** ^1^ Key Laboratory of Ministry of Education for Conservation and Utilization of Special Biological Resources in the Western China, Ningxia University, Yinchuan, China; ^2^ School of Life Science, Ningxia University, Yinchuan, China; ^3^ Department of Beijing National Biochip Research Center sub-center in Ningxia, General Hospital of Ningxia Medical University, Yinchuan, China; ^4^ Department of Anatomy and Cell Biology, University of Iowa, Carver College of Medicine, Iowa City, IA, United States; ^5^ Analysis and Testing Center, Ningxia University, Yinchuan, China

**Keywords:** Heme oxygenase-1, ferroptosis, macrophage, *Mycobacterium tuberculosis*, Bacillus Calmat and Guerin

## Abstract

Macrophages are the host cells and the frontline defense against *Mycobacterium tuberculosis* (Mtb) infection, and the form of death of infected macrophages plays a pivotal role in the outcome of Mtb infections. Ferroptosis, a programmed necrotic cell death induced by overwhelming lipid peroxidation, was confirmed as one of the mechanisms of Mtb spread following infection and the pathogenesis of tuberculosis (TB). However, the mechanism underlying the macrophage ferroptosis induced by Mtb infection has not yet been fully understood. In the present study, transcriptome analysis revealed the upregulation of heme oxygenase-1 (*HMOX1*) and pro-ferroptosis cytokines, but downregulation of glutathione peroxidase 4 (*GPX4*) and other key anti-lipid peroxidation factors in the peripheral blood of both patients with extra-pulmonary tuberculosis (EPTB) and pulmonary tuberculosis (PTB). This finding was further corroborated in mice and RAW264.7 murine macrophage-like cells infected with *Bacillus Calmette-Guerin* (BCG). A mechanistic study further demonstrated that heme oxygenase-1 protein (HO-1) regulated the production of reactive oxygen species (ROS) and iron metabolism, and ferroptosis in BCG-infected murine macrophages. The knockdown of *Hmox1* by siRNA resulted in a significant increase of intracellular ROS, Fe^2+^, and iron autophagy-mediated factor Ncoa4, along with the reduction of antioxidant factors Gpx4 and Fsp1 in macrophages infected with BCG. The siRNA-mediated knockdown of *Hmox1* also reduced cell survival rate and increased the release of intracellular bacteria in BCG-infected macrophages. By contrast, scavenging ROS by N-acetyl cysteine led to the reduction of intracellular ROS, Fe^2+^, and Hmox1 concentrations, and subsequently inhibited ferroptosis and the release of intracellular BCG in RAW264.7 cells infected with BCG. These findings suggest that HO-1 is an essential regulator of Mtb-induced ferroptosis, which regulates ROS production and iron accretion to alter macrophage death against Mtb infections.

## 1 Introduction

Tuberculosis (TB) is a chronic disease caused by the infection of *Mycobacterium tuberculosis* (Mtb), which remains a major public health burden in many developing countries, with approximately 2 billion latent infections and 9.87 million new cases in 2020 (WHO). A recent study confirmed that a co-infection of COVID with Mtb could worsen the COVID-19 infection (Visca et al., 2021). Although considerable efforts have been made in combating Mtb infections and TB disease, the elusive pathogenesis in the development of TB leads to the difficulty and challenges in the prevention and treatment of this ancient disease (Sheedy and Divangahi, 2021). There is not an effective TB vaccine currently available for adults, and clinical treatments for TB are largely dependent on antibiotics such as rifampin. It is therefore a necessity to better understand the pathogenesis of TB disease.

Macrophages are the host cells and the frontline defense of Mtb infections (Korb et al., 2016). The outcome of the host–pathogen interaction between macrophages and Mtb is certainly critical for the development of TB (Xu et al., 2014). In this context, intracellular Mtb either can be eradicated through macrophage apoptosis (Lee et al., 2009) and autophagy (Alam et al., 2017), or can persistently survive and grow in macrophages, and induce macrophage necrosis and spread infection to other cells by evolving an immune escape mechanism (Liu et al., 2017; Pajuelo et al., 2018; Zhai et al., 2019). Therefore, understanding the molecular mechanism of Mtb-induced macrophage deaths, particularly macrophage necrosis, may enable us to uncover novel targets for host-directed therapy (HDT) of TB by altering macrophage death in response to Mtb infections.

In addition to necroptosis and pyroptosis, the two most studied forms of Mtb-induced macrophage necrosis, ferroptosis is another form of programmed cell death (PCD) that is a type of necrosis dependent on iron (Dixon et al., 2012; Amaral et al., 2019). Interestingly, macrophage necrosis induced by Mtb infection shared the typical characteristics of ferroptosis (Shastri et al., 2018; Amaral et al., 2021; Baatjies et al., 2021). In this regard, an external stress such as Mtb infection could elevate intracellular levels of Fe^2+^ and reactive oxygen species (ROS) to trigger the Fenton reaction, and result in the production of large amounts of hydroxyl radicals, sequentially impair glutathione peroxidase 4 (GPX4) activity and anti-lipid peroxidation capacity, and ultimately lead to overwhelming lipid peroxidation of intracellular membrane phospholipids and cell disintegration and death (Dixon et al., 2012; Li et al., 2020). Indeed, the ferroptosis inhibitor could effectively reduce Mtb-induced macrophage necrosis and bacterial load in a mouse infection model (Amaral et al., 2019). These findings suggest that intracellular ROS and lipid peroxidation are essential for ferroptosis in response to Mtb infections, implying that oxidation-related ferrous ion imbalance is involved in cell ferroptosis death; however, the molecular mechanism underpinning the ferrous ion production in Mtb-induced macrophage ferroptosis has not been completely elucidated.

Heme oxygenase 1 protein (HO-1) is encoded by *HMOX1* gene, which is an important stress-responsive enzyme highly expressed in lungs, which catalyzes to degrade heme to Fe^2+^, carbon monoxide (CO), biliverdin, and bilirubin, and is essential in the balance of intracellular Fe^2+^ and ROS (Araujo et al., 2012). Moreover, HO-1 is also considered as one of the biomarkers for the diagnosis of TB in clinical settings (Rockwood et al., 2017; Yong et al., 2019; Uwimaana et al., 2021; Yang et al., 2022); whether it plays a role in the regulation of Mtb-induced ferroptosis in macrophages, however, has yet been investigated.

In the present study, the involvement of *HMOX1* in macrophage ferroptosis in response to a mycobacterial infection was interrogated by transcriptome analysis of peripheral blood in TB patients, and its mechanism in ferroptosis was further investigated in mice and macrophage-like RAW264.7 cells by infection of *Bacillus Calmette-Guerin* (BCG). Our results demonstrate that HO-1 is a negative regulator of murine macrophage ferroptosis in response to BCG infections, in part through a mechanism by which HO-1 inhibits the intracellular ROS production and iron accretion, but induces Gpx4 expression in murine macrophages infected by BCG.

## 2 Materials and methods

### 2.1 Mice

C57BL/6 mice were purchased from Gempharmatech Co., Ltd (Jiang Su, China). Experiments using mice were approved by the Laboratory Animal Welfare Ethics Review Committee of Ningxia University (NXU-2018-011). Sixteen mice were randomly divided into two groups, PBS control and BCG infection. Mice were housed in specific pathogen-free conditions in a 12-h light/dark cycle with *ad libitum* access to food and water. All animal studies were conducted at the Laboratory Animal Center of Ningxia University (Yinchuan, China).

### 2.2 Bacteria and infection

*Mycobacterium tuberculosis* attenuated strain BCG was purchased from Chengdu Institute of Biological Products Ltd (Chengdu, China). The lyophilized bacteria preparation was dissolved and washed with PBS, and subsequently gently sonicated to disrupt bacterial clumps for single-cell suspension in DMEM or PBS. The bacterial suspension was aliquoted and stored at −20°C, and was used within 2 weeks after the preparation. For bacterial infection in mice, mouse was infected with BCG in 100 µl at a dose of 5 × 10^6^ colony-forming units (CFU)/mouse *via* tail vein injection. The lung tissues were harvested for analysis at 30 days post-infection.

### 2.3 Chemicals and small interfering RNA

GPX4 inhibitor RAS-selective lethal 3 (RSL3) (#HY-100218A) and ROS scavenger N-acetylcysteine (NAC) (#HY-134495) were purchased from MedChemExpress (Shanghai, China), and were dissolved in DMSO. H_2_O_2_ (#7722-84-1) was from MERCK (Germany). Three small interfering RNAs (siRNAs) to murine *Hmox1* mRNA sequence (Gene ID: NM_010442.2), si*-Hmox1*-27 5’-GTTTCCGCATACAACCAGTGA-3’, si*-Hmox1*-193 5’-UUGGAUGUGUACCUCCUUGTT-3’, and si*-Hmox1*-672 5’-GCUCUAUCGUGCUCGAAUGAA-3’, were designed using *In vivo*Gen-siRNA Wizard Software 3.1 (https://www.invivogen.com/sirnawizard/design_advanced.php, *In vivo*Gen, California, USA). Control siRNA 5’-UUCUCCGAACGUGUCACGUTT-3’ served as the scramble siRNA. The siRNAs were synthesized by GenePharma (Shanghai, China).

### 2.4 Cell culture, *in vitro* BCG infection, and siRNA transfection

RAW264.7 murine monocyte/macrophage-like cells were cultured in DMEM containing 10% calf serum at 37°C in 5% CO_2_ atmosphere. For bacterial infection, RAW264.7 cells were seeded in a six-well plate at a density of 2 × 10^5^/well and cultured for 16 h. Cells were then pretreated with NAC for 1 h or transfected with siRNA for 12 h prior to be infected with bacteria at a multiplicity of infection (MOI) of 1, 5, or 10. The cells were harvested for analysis at 24 h post-infection. siRNA was transfected with Lipofectamine RNAiMAX per manufacturer’s instructions (ThermoFisher Scientific, Waltham, MA, USA). To test the efficiency of siRNA-mediated knockdown of protein of interest, total proteins of transfected cells were extracted at 24 h post-transfection and were analyzed by Western blotting assay.

### 2.5 Western blotting

Total proteins of cells were lysed with RAPI buffer containing protease inhibitor (#P0033, Beyotime, Shanghai, China). The protein concentration of soluble fraction of cell lysates was determined using the Pierce™ BCA agents (#23225, ThermoFisher). The protein samples were separated by SDS-PAGE prior to being transferred to PVDF membranes. The PVDF membrane was then blocked with 5% fat-free milk for 1 h at room temperature (RT), before it was incubated with primary antibodies to protein of interest for overnight at 4°C with shaking (45 rpm). The membrane was then washed 3 × 10 min in TBST solution containing 0.2% Tween-20 prior to being incubated in appropriate horseradish peroxidase (HRP)-conjugated secondary antibody for 1 h at RT. The specific binding of protein was developed in Western Lightning^®^ Plus-ECL (#NEL105001EA, PerkinElmer) and visualized in Amersham Imager 600 (Cytiva, USA). The abundance of protein was semi-quantified with ImageJ 1.52a as described elsewhere (Pillai-Kastoori et al., 2020). Beta-actin or tubulin served as internal loading control; GraphPad was used to calculate “Mean ± SD” and statistical analysis. The antibodies used in this study are listed in **Supplementary Table S1**.

### 2.6 Measurement of apoptosis and necroptosis

Cells were treated with the Annexin V/PI Apoptosis Detection kit (KGA108-1, keygen Biotech, Nan Jing, China). Cells were washed with PBS, followed by being centrifuged at 2,000 rpm for 5 min, and the medium supernatant was discarded. The resulting cell pellet was washed twice with PBS by centrifugation. A total of 1–5 × 10^5^ cells were suspended in 500 µl of binding buffer before 5 μl of Annexin V-FITC and 5 μl of propidium iodide were added consecutively. The cell suspension was incubated for 5 min at room temperature in the dark. The above samples were detected using a flow cytometer. The processed samples were detected by flow cytometry.

### 2.7 Measurement of cell death

Cell viability was measured by Trypan Blue staining assay. 0.4% Trypan Blue solution was added to a single-cell suspension at a ratio of 1:9 in volume. Live/dead cells were read using Invitrogen Countess 3 (ThermoFisher) and the percentage of live cells was calculated.

### 2.8 Bacterial colony-forming unit count

RAW264.7 macrophages were preincubated in medium containing 2.0 mM NAC for 1 h, or transfected with siRNA*-Hmox1* for 24 h, followed by incubating with BCG at an MOI of 5 for 1 h. The cells were then rinsed with medium to remove unattached bacteria and cultured with fresh medium for an additional 24 h. The culture medium was then collected for accessing the release of intracellular bacteria due to cell necrotic death including ferroptosis by spreading series diluted medium in 7H10 agar plates and incubated at 37°C for 21 days. The number of colonies on the plates was counted as CFU numbers. The final bacterial CFU number was calculated by multiplying the dilution factor and the count on plates.

### 2.9 Intracellular iron measurement

The content of intracellular ferrous iron (Fe^2+^) was measured using the Iron Assay kit (#ab83366, Abcam). Only ferrous (Fe^2+^) can bind to the iron probe to form a stable-colored complex that can be accessed by reading an absorption peak at a wavelength of 539 nm. Solutions of standard curve and reaction were prepared in accordance with the manufacturer’s instructions.

### 2.10 Intracellular ROS detection

Intracellular ROS detection was measured with the CellROX™ Deep Red kit (#C10491, ThermoFisher). CellROX™ Red is a fluorescent probe for measuring ROS in living cells. CellROX™ Red emits a stable bright red fluorescence upon an oxidation by ROS. The maximum absorption/emission wavelength is approximately 485/520 nm.

### 2.11 Mitochondrial fluorescent staining

Live cell mitochondria were stained with CellLight™ Mitochondria-GFP BacMam 2.0 (#C10508, ThermoFisher). CellLight™ Mitochondria-GFP was added to the cell cultures, and cells were continuously incubated for an additional 16 h at 37℃. The mitochondrial fluorescence was then observed in a laser confocal microscope (SP5, Leica, Germany).

### 2.12 Lipid peroxidation assay

An Image-iT^®^ Lipid Peroxidation Kit (#C10445, ThermoFisher) was used to assay cellular lipid peroxidation. The BODIPY^®^ 581/591 C11 probe binds to polyunsaturated fatty acids in the cell membrane. Due to the oxidation of fatty acids, the absorption peak of the probe shifts from 590 nm (red) to 510 nm (green); the change in value of the absorption peaks at different wavelengths was calculated using the arithmetic mean. For immunofluorescence assays, live cells were incubated with the BODIPY^®^ 581/591 C11 reagent for 30 min. Cell membrane lipid peroxidation was then respectively observed under excitation (581/488 nm)/emission (591/510 nm) conditions. The staining was observed and imaged under a laser scanning confocal microscope (SP5, Leica, Germany).

### 2.13 Immunocytofluorescence staining

RAW264.7 cells were seeded on collagen pre-coated sterile cover slides. Cells were fixed in 4% paraformaldehyde for 30 min, washed in PBS for 3× 5 min, and penetrated with 0.1% Triton X-100 (PBST) for 15 min at RT. Cells on slides were then blocked with 5% BSA for 1 h at RT, followed by incubating in primary antibody overnight at 4°C. Slides were then washed 3× 5 min in PBST before they were incubated with appropriate fluorescent secondary antibody with light proof for 1 h at RT. Cell nuclei were counterstained with DAPI. Images were acquired under a laser scanning confocal microscope (SP5, Leica, Germany).

### 2.14 Immunohistochemistry staining

The lung tissues were fixed in 4% paraformaldehyde and embedded in paraffin. Four-micrometer-thick sections were cut with a Leica RM2135 Microtome (Germany). After xylene deparaffination, rehydration, and antigen retrieval, the sections were blocked in 10% donkey serum blocking buffer. Slides were then incubated in primary antibodies overnight at 4°C, followed by being incubated with HRP-conjugated secondary antibody for 1 h at RT. The antigen–antibody binding was detected with DAB substrate solution. The sections were counterstained with hematoxylin for nuclei. The staining was observed and imaged under the microscope (BA400Digital, Motic, USA). Immunohistochemical (IHC) images were analyzed using ImageJ-IHC profiler for semi-quantification as described elsewhere (Crowe and Yue, 2019). GraphPad 8.0.1 was used for statistical analysis.

### 2.15 Transmission electron microscopy test

Cell pellets were resuspended in 0.5% glutaraldehyde fixative and incubated for 10 min at 4°C. Cells were re-pelleted and treated with 1% osmium tetroxide. The cell samples were then dehydrated with a low to high concentration of acetone prior to be embedded in epoxy resin. Fifty-nanometer-thick sections were prepared using Ultramicrotome EM UC7 (Leica, Germany). Samples were stained with uranyl acetate and lead citrate. Transmission electron microscopy was used to observe cell morphology.

### 2.16 GEO data analysis

The GSE83456 dataset is sourced from the Gene Expression Omnibus (GEO) database. The Ethic Committee of Human Research at General Hospital of Ningxia Medical University approved the protocol of human study. The dataset includes peripheral blood transcriptome sequencing information for three groups, namely, 30 healthy control subjects (Control), 30 individuals with extra-pulmonary tuberculosis (EPTB), and 30 patients with pulmonary tuberculosis (PTB). The demographics of TB patients whose blood samples were collected and used in this study are listed in Supplementary Table S2. The dataset was normalized by Log2 transformation in R software 3.4.1. Subsequently, the gene IDs were converted to gene names. The significance of the three groups of datasets was analyzed in GraphPad 8.0.1 using one-way ANOVA followed by Tukey’s multiple comparisons test.

### 2.17 ELISA testing of clinical samples

The Human Heme Oxygenase 1 (HO-1) ELISA Kit (#ab207621, Abcam) was used to detect HO-1 protein levels in the peripheral blood of clinical TB patients (*n* = 59) and healthy individuals (*n* = 26). The Hemin Assay Kit (#ab65332, Abcam) was employed for determining the concentration of H in the serum of TB patients (*n* = 57) and healthy individuals (*n* = 26). The measurement was performed per the manufacturer’s instructions. The collection and processing of human peripheral blood samples passed the approval of the Medical Research Ethics Review Committee at General Hospital of Ningxia Medical University General Hospital (NO. 2020-410).

### 2.18 Statistics

All graphical data are expressed as mean ± SD. Graphical data and statistical analyses were processed with GraphPad Prism 8.0.1. Comparisons between the two groups were carried out using an unpaired two-tailed *t*-test and triple comparisons were statistically analyzed using one-way ANOVA. Charts and legends provide the number of independent experiments and the results of a representative experiment.

## 3 Results

### 3.1 Transcriptome analysis of peripheral blood revealed differential expression of ferroptosis-related genes in patients with tuberculosis

**Figure 1:**
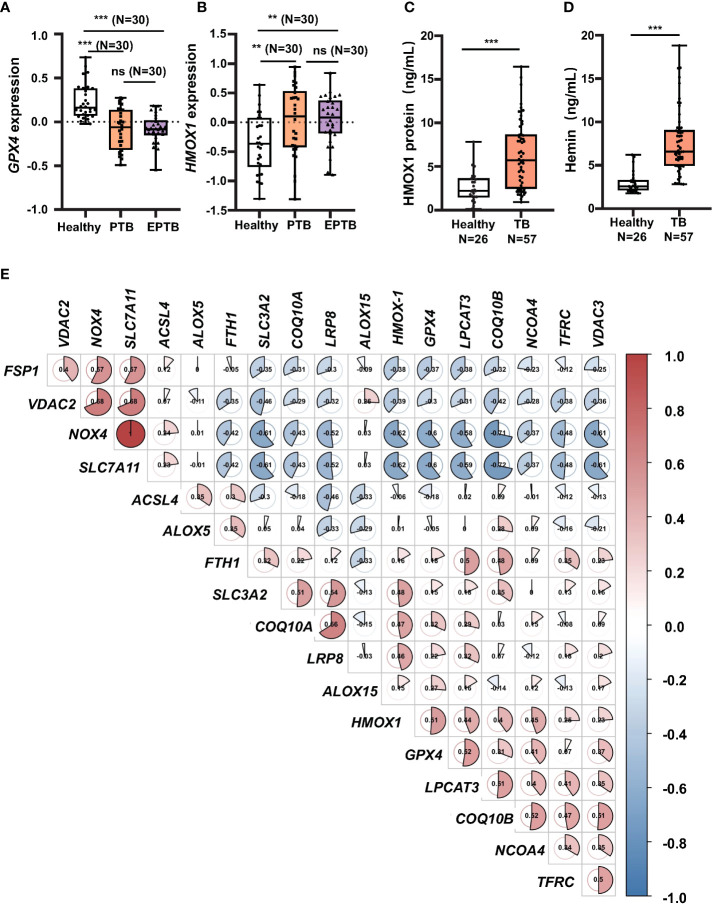
Increase of *HMOX1* and decrease of *GPX-4* in peripheral blood of TB patients. Peripheral blood from healthy individuals (*N* = 30), patients with pulmonary TB (PTB, *N* = 30), and patients with extra-pulmonary TB (EPTB, *N* = 30) was collected for transcriptome analysis using GEO database GSE83456 (a transcriptome sequencing dataset of peripheral blood from TB patients). **(A, B)** The differential expression of transcript of the anti-lipid peroxidation factor *GPX4* and **(B)** the oxidative stress regulator *HMOX1* in the peripheral blood of TB patients. **(C)** More abundant circulating HO-1 protein in blood of patients with TB in comparison with healthy subjects as determined by ELISA. **(D)** Hemin content in peripheral blood of patients with TB in comparison with healthy subjects as determined by the Hemin Assay Kit. **(E)** Plotting CorrPlot map in Hiplot (https://hiplot-academic.com/) showed the correlation of *HMOX1* transcript with transcripts of ferroptosis markers in patients with PTB analyzed by Spearman. The color in the CorrPlot map represents the correlation coefficient, darker color represents stronger correlations. Red represents positive correlation while blue represents negative correlation. Cluster analysis using the ward.D2 method in Hiplot. Data were processed using GraphPad Prism 8.0.1 software and ImageJ 1.52.a. Unpaired *t*-test was used to analyze the differential changes of two groups. One-way ANOVA and Tukey’s multiple comparisons test was used to analyze the differential changes of multiple groups. Data represented mean ± SD; significant differences were indicated with asterisks (**p < 0.01; ***p < 0.001). ns, no statistical difference.

To investigate whether ferroptosis is involved in the pathogenesis of TB development in clinic, peripheral blood of healthy individuals (Control, *N* = 30), patients with PTB (*N* = 30), and patients with EPTB (*N* = 30) was collected for transcriptome analysis, and compared with the publicly available GEO database (GSE83456). Collated datasets were processed using log2 normalization, and the ID_REF was converted to gene symbols. The transcriptome analysis demonstrated that anti-lipid peroxidation factor *GPX4*, a major scavenger of phospholipid hydroperoxides, and the key regulator of ferroptosis, was significantly downregulated (**Figure 1A**), but anti-oxidative stress factor *HMOX1* was upregulated (**Figure 1B**) in both PTB and EPTB patients, compared to healthy control cohorts. Moreover, the increased level of *HMOX1* transcript was in line with the more abundant HO-1 protein in peripheral blood from TB patients (6.333 ng/ml, *N* = 57) compared to healthy individuals (2.58 ng/ml, *N* = 26), as determined by ELISA (**Figure 1C**). Heme B is a substrate for the enzymatic reaction of HO-1, and forms a chlorine-containing porphyrin, namely, Hemin in blood. Hemin is a potent inducer of HO-1 and plays a certain extent of anti-infective roles in the body (Kim et al., 2021). Hemin content in peripheral blood was significantly higher in TB patients (0.85 ± 0.44 ng/ml, *N* = 57) than healthy individuals (0.29 ± 0.26 ng/ml, *N* = 26) (**Figure 1D**). This result was in line with the concentration of HO-1 protein in peripheral blood TB patients.

In order to reveal a correlation between *HMOX1*, an oxidative stress signature, and ferroptosis regulators, a CorrPlot map was constructed using Spearman rank correlation (**Figure 1D**). The CorrPlot map showed that the *HMOX1* expression was negatively associated with the level of circulating *GPX4*, suggesting that HO-1 might play a regulatory role in the ferroptosis in TB pathogenesis. In addition, transcriptome analysis also unraveled the differential expression of other ferroptosis genes in peripheral blood of TB patients (**Supplementary Figure S1**). Among them, the transcripts of *ACSL4*, *LPCAT3*, *ALOX5*, *COQ10A*, *VDAC2*, *NOX4*, *xCT*, and *FTH1* genes were increased in peripheral blood of both patients with PTB and EPTB (**Supplementary Figures S1A, D, E, G, I, J, N**), while *LPCAT3* and *COQ10B* transcripts were only increased in PTB but not EPTB patients (**Supplementary Figures S1B, F**), as compared with the healthy individuals. There was no statistical significance in differential expression of transcripts *ALOX15*, *VDAC3*, *SLC3A2*, *NCOA4*, and *LRP8* genes (**Supplementary Figures S1D, K, L, M**). These data clearly evidence that ferroptosis is involved in the TB pathogenesis.

### 3.2 BCG induced ferroptosis in macrophages

**Figure 2:**
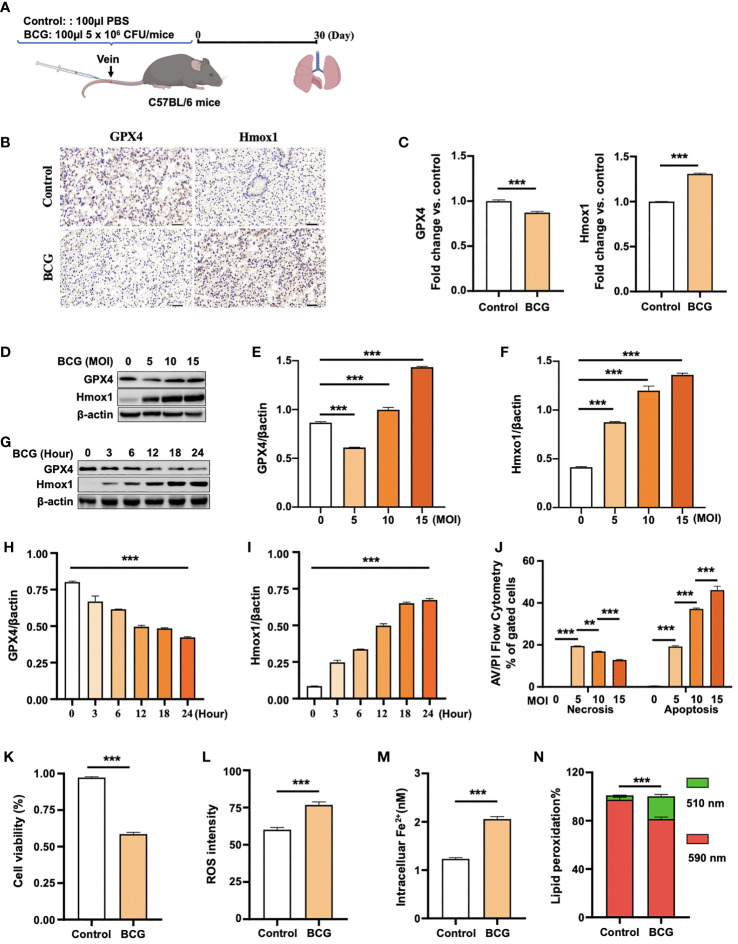
BCG induces ferroptosis in macrophages. **(A)** Schematic diagram shows the infection of mice with BCG *via* tail vein injection. The infected mice are analyzed at 30 days post-infection (DPI). **(B, C)** C57BL/6 mice were injected with 5 × 10^6^ CFU BCG in 100 µl *via* the tail vein and the lungs were harvested for evaluating the expression of Gpx4 and HO-1 proteins at 30 DPI by immunohistochemical (IHC) assay **(B)** and semi-quantified by ImageJ-IHC Profiler **(C)**. Lungs of mice infected with BCG showed less and more abundant Gpx4 and HO-1 proteins compared to the uninfected controls, respectively. Bar represents 500 μm in **(B) (D-F)** RAW264.7 murine macrophage-like cells were infected with BCG at indicated MOI for 24 h, and the abundance of Gpx4 and HO-1 proteins was examined by Western blotting assay. The representative blots **(D)** and semi-quantification of Gpx4 **(E)** and HO-1 **(F)** showed a dose-dependent induction of Gpx4 and HO-1 in this type of cells, except an inhibition of Gpx4 expression in cells infected with a low dose of BCG at an MOI of 5. **(G–I)** The representative blots **(D)** and semi-quantification of Gpx4 **(H)** and HO-1 **(I)** demonstrated a time-dependent inhibition of Gpx4 and induction of HO-1 in RAW264.7 infected with BCG at an MOI of 5 for a 24-h time period. **(J)** Induction of cell necrosis and apoptosis in RAW264.7 cells at 24 h post-infection of BCG at the indicated MOI determined by using Annexin V/PI in flow cytometry. **(K)** Cell viability assay showed that the infection of BCG decreased the viability of RAW264.7 cells at an MOI of 5 for 24 has detected by Trypan Blue assay. **(L)** The infection of BCG induced the production of intracellular ROS in RAW264.7 cells at an MOI of 5 for 24 has detected by flow cytometry assay. **(M)** The infection of BCG increased the concentration of intracellular Fe^2+^ in RAW264.7 cells at an MOI of 5 for 24 has detected using iron ion probes. **(N)** The infection of BCG induced lipid peroxidation in RAW264.7 cells at an MOI of 5 for 24 h as determined by BODIPY 581/591 C11 assays. Upon oxidation, its excitation of Red/590 nm shifts to 510 nm (Green). The ratio of Green/Red cells in the BCG-infected cells was 17.22%, while the uninfected cells was 3.52%. Data obtained from three independent experiments were processed using GraphPad Prism 8.0.1 software and ImageJ 1.52.a. Unpaired *t*-test was used to analyze the differential changes of the two groups. One-way ANOVA and Tukey’s multiple comparisons test was used to analyze the differential changes of multiple groups. Data represented mean ± SD from three independent experiments; significant differences are indicated with asterisks (**p < 0.01; ***p < 0.001).

To test whether the infection of Mycobacteria could induce ferroptosis *in vivo*, 8-week ICR mice were infected with the Mtb-attenuated strain BCG at a dose of 5 × 10^6^ CFU/mouse in 100 µl volume *via* tail vein injection, and the ferroptosis was assessed by the expression of Gpx4 and HO-1 proteins in lungs at 30 days post-infection (**Figure 2A**). IHC staining assay showed a reduced Gpx4 protein, the signature of cell ferroptosis, and an increased HO-1 protein abundance in lungs of BCG-infected mice compared to the control group (**Figures 2B, C**), which was in accordance with the transcriptomic findings in peripheral blood of TB patients (**Figure 1** and **Supplementary Figure S1**).

**Figure 3:**
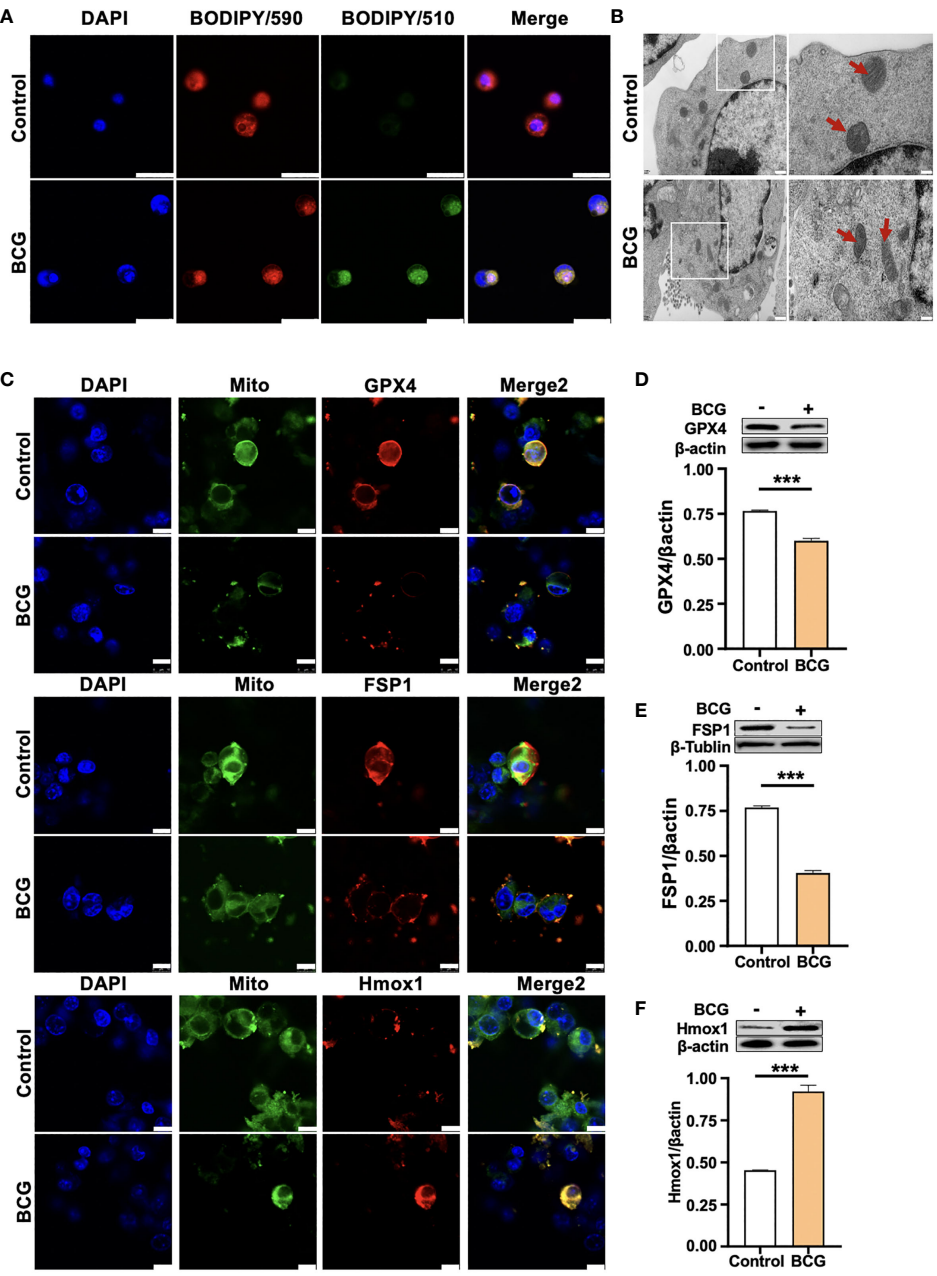
HO-1 is involved in the BCG-induced macrophage ferroptosis. **(A)** Representative images of BODIPY 581/591 C11-labeled lipoxidation of polyunsaturated fatty acids. BCG-infected RAW264.7 cells showed a strong positive lipid peroxidation (Green/510 nm) compared to the uninfected cells. **(B)** Representative images of transmission electron microscopy showed mitochondrial membrane ridge breaks (arrows) in RAW264.7 macrophages infected by BCG; the right panel shows the enlarged image of the boxed area in its corresponding image in the left panel. **(C)** Representative immunofluorescence images of Gpx4 (top panels), Fsp1 (middle panels), and HO-1 (bottom panels) showed the decrease of anti-ferroptotic markers Gpx4 and Fsp1, but increased pro-ferroptotic marker HO-1 in BCG-infected cells. Cell mitochondria were labeled with CellLight™ Mitochondria-GFP (green), which were reduced in macrophages following the BCG infection. **(D–F)** Representative blots and semi-quantitative analysis of Gpx4, Fsp1, and HO-1 proteins of RAW264.7 cells. Statistical analysis of data performed using GraphPad Prism 8.0.1 software and ImageJ 1.52.a. Cell nuclei were counterstained with DAPI. Data obtained from three independent experiments were processed using GraphPad Prism 8.0.1 software and ImageJ 1.52.a. Unpaired *t*-test was used to analyze the differential changes of two groups. Data are presented as mean ± SD from three independent experiments (***p < 0.001; *n* = 3). Bars, 500 nm in the right panel and 200 nm in the left panel of A, 25 μm in B, and 10 μm in C.

Given the Mtb-infected macrophage death in TB pathogenesis, we next investigated whether the BCG infection could induce ferroptosis in RAW264.7 murine macrophage-like cells. Immunoblotting assay demonstrated a dose-dependent induction of Gpx4 and HO-1 in this type of cells, except for an inhibition of Gpx4 expression in cells infected with a low dose of BCG at an MOI of 5 (**Figures 2C–F**). Interestingly, the inhibition of Gpx4 and induction of Hmox1 was in a time-dependent manner, when cells were infected with BCG at an MOI of 5 for the 24-h time period (**Figures 2G, H, I**). Annexin V/PI flow cytometry revealed a dose-dependent reduction of cell necrosis but an increase of apoptosis in RAW264.7 cells at 24 h post-infection of BCG at an MOI range of 5 to 15 (**Figure 2J** and **Supplementary Figure S2A**). As expected, the infection of BCG significantly reduced cell viability (**Figure 2K**) and increased the production of intracellular ROS (**Figure 2L**; **Supplementary Figure S2B**) and the intracellular level of Fe^2+^ (**Figure 2M**). Importantly, the infection of BCG increased the fraction of cells with lipid peroxidation as determined by BODIPY 581/591 C11 assays (**Figures 2N** and **3A**; **Supplementary Figure S2C**). The ratio of lipid peroxidation cell (Green)/normal cell (Red) was 17.22% in the BCG-infected cells, while it was 3.52% in uninfected cells (**Figure 2K**). Transmission electron microscope (TEM) observation of BCG-infected RAW264.7 macrophages showed increased mitochondrial membrane ridge breaks compared to uninfected cells (**Figure 3B**). Mechanistic study by immunocytochemistry (ICC) (**Figure 3C**) and Western blotting (**Figures 3D–F**) demonstrated a decrease of anti-ferroptosis regulators Gpx4 and Fsp1, along with the decrease of cell mitochondria staining (**Figure 3C**), but an increase of pro-ferroptosis marker HO-1 in BCG-infected cells compared with uninfected cells. These observations were further corroborated by a molecular study using Western blotting assay (**Figures 3D–F**). In addition, the expression of anti-ferroptosis molecules Alox5 (**Supplementary Figure S3B**), Vdca2 (**Supplementary Figure S3C**), and Ncoa4 (**Supplementary Figure S3D**) was also inhibited, while pro-ferroptosis factor xCT (**Supplementary Figure S3E**) was increased in cells infected with BCG, relative to the uninfected controls. These results indicated that the infection of BCG could induce ferroptosis of RAW264.7 cells, and the increase of intracellular ROS production and the alteration of HO-1 were part of the underlying mechanism of BCG-induced ferroptosis.

### 3.3 Intracellular ROS contributes to BCG-induced ferroptosis in RAW264.7 macrophages

**Figure 4:**
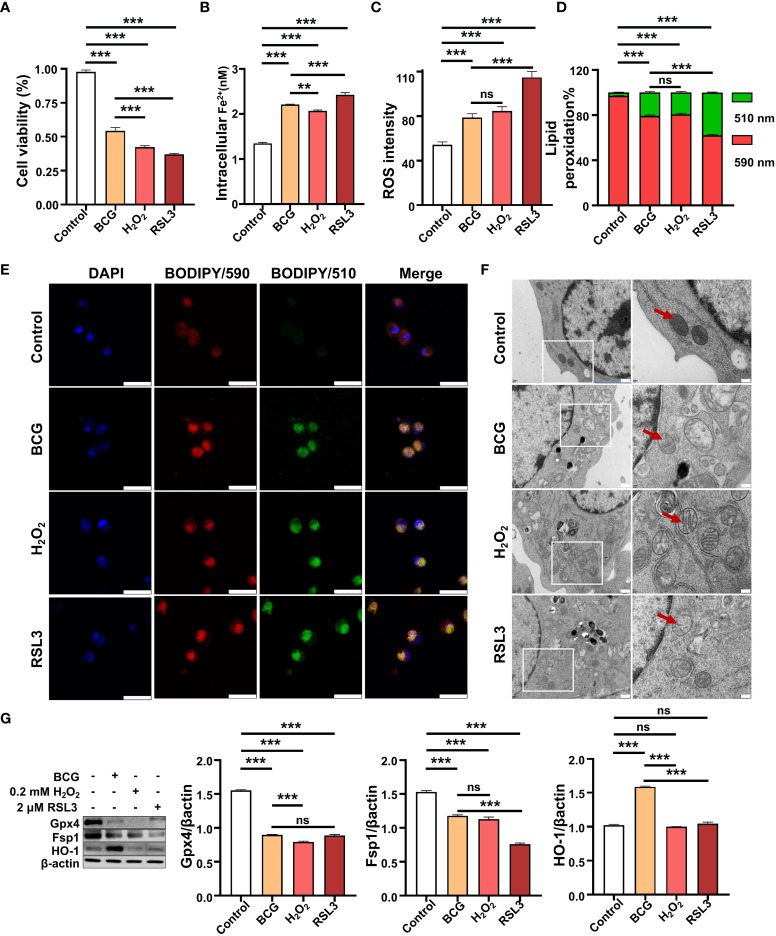
BCG induces intracellular ROS production and ferroptosis in RAW264.7 macrophages. **(A–D)** The viability **(A)**, intracellular Fe^2+^ **(B)**, intracellular ROS **(C)**, and lipid peroxidation **(D)** of RAW264.7 macrophages in response to BCG infection (MOI 5), ferroptosis agonist RSL3 (2.0 µM), and H_2_O_2_ (0.2 mM), major participants of the Fenton response at 24 h post-treatments, as determined by Trypan Blue assay, iron ion probes, flow cytometry, and BODIPY 581/591 C11 assays, respectively. **(E)** Representative fluorescence images of BODIPY 581/591 C11-labeled lipoxidation of polyunsaturated fatty acids in RAW264.7 macrophages infected with BCG (the second panel from top), treated with H_2_O_2_ (the second panel from bottom), and RSL3 (bottom panel). Cell nuclei were counterstained with DAPI. **(F)** Representative images of transmission electron microscopy showed mitochondrial membrane ridge breaks (arrows) in RAW264.7 macrophages infected with BCG (the second panel from top), treated with H_2_O_2_ (the second panel from bottom) and RSL3 (bottom panel). The right panel shows the enlarged image of the boxed area in its corresponding image in the left panel. **(G)** Representative blots and semi-quantitative analysis of Gpx4, Fsp1, and HO-1 proteins of RAW264.7 cells treated with the indicated conditions. The infection of BCG increased intracellular Fe^2+^ level and induced ROS (H_2_O_2_) production and lipid peroxidation and ferroptosis in macrophages, by inhibiting Gpx4 and Fsp1 but inducing HO-1 expression. Data obtained from three independent experiments were processed using GraphPad Prism 8.0.1 software and ImageJ 1.52.a. One-way ANOVA was used to analyze the differences between groups. All values are presented as mean ± SD (**p < 0.01, and ***p < 0.001; *n* = 3). Bars, 500 nm in the right panel and 200 nm in the left panel of E, 25 μm in F. ns, no statistical difference.

Next, we sought whether the intracellular ROS contributed to ferroptosis in RAW264.7 cells in response to BCG infections. In order to investigate the effect of ROS in ferroptosis, H_2_O_2_, a major ROS source, and RSL3, one of the most common inhibitors of GPX4 and inducer of ferroptosis, separately served as positive controls. Optimization experiments showed that the highest inhibition of the ferroptosis regulator Gpx4 was found in RAW264.7 cells exposed to H_2_O_2_ and RSL3 at concentrations of 0.2 mM and 2 mM, respectively (**Supplementary Figures S4A, B**). These optimized concentrations were therefore used in further experiments of this report. Indeed, similar to treatments of H_2_O_2_ and RSL3, the infection of BCG also displayed effects of inhibition of cell viability (**Figure 4A**), inductions of intracellular Fe^2+^ (**Figure 4B**) and ROS production (**Figure 4C** and **Supplementary Figure S4C**), and increase of lipid peroxidation (**Figures 4D, E** and **Supplementary Figure S4D**) in RAW264.7 macrophages at an MOI of 5. As a consequence, these treatments induced mitochondrial membrane ridge breaks (arrows) in RAW264.7 macrophages as determined by electron microscope observation (**Figure 4F**). Molecular analysis further demonstrated that the infection of BCG significantly inhibited the expression of Gpx4 and Fsp1 proteins, but increased HO-1 expression (**Figure 4G**). Of note, the treatments of H_2_O_2_ or RSL3 failed to increased HO-1 protein, although these treatments inhibited Gpx4 and Fsp1 in RAW264.7 cell as seen in BCG-infected cells (**Figure 4G**). The infection of BCG is comparable to intracellular ROS production and cell peroxidation, and to the inhibition of the expression of Gpx4 and Fsp1 with that of 0.2 mM of H_2_O_2_, although the effect was to a lesser extent compared to that of 2.0 mM RSL in RAW264.7 cells. These results suggest that HO-1-mediated ROS production may play a major role in the regulation of ferroptosis in macrophages against Mtb infections.

### 3.4 ROS scavenger NAC reduces BCG-induced macrophage ferroptosis

**Figure 5:**
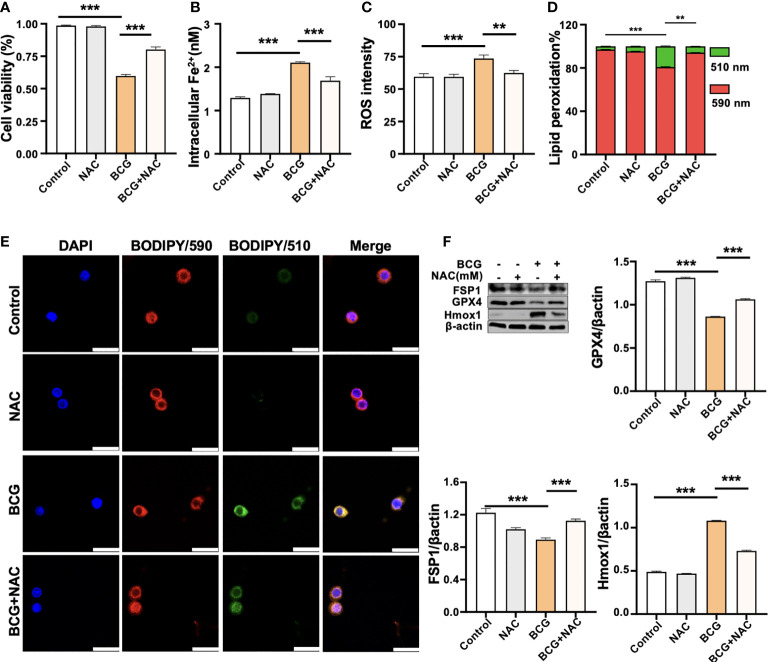
ROS scavenger NAC reduces BCG-induced macrophage ferroptosis. RAW264.7 macrophages were preincubated in medium containing 2.0 mM NAC for 1 h prior to being infected with BCG at an MOI of 5 for 24 h, before they were harvested for analysis. **(A–D)** The viability **(A)**, intracellular Fe^2+^ **(B)**, intracellular ROS **(C)**, and lipid peroxidation **(D)** of RAW264.7 macrophages treated with the indicated conditions, as determined by Trypan Blue assay, iron ion probes, flow cytometry, and BODIPY 581/591 C11 assays, respectively. **(E)** Representative fluorescence images of BODIPY 581/591 C11-labeled lipoxidation of polyunsaturated fatty acids in RAW264.7 macrophages of the indicated conditions showed the reduction of BCG-induced lipoxidation in cells pretreated with NAC. Cell nuclei were counterstained with DAPI. **(F)** Representative blots and semi-quantitative analysis of Gpx4, Fsp1, and HO-1 proteins of RAW264.7 cells treated with the indicated conditions. The NAC pretreatment increased Gpx4 and Fsp1 expression, but decreased Hmox1 protein in BCG-infected cells. Data obtained from three independent experiments were processed using GraphPad Prism 8.0.1 software and ImageJ 1.52.a. All values are presented as mean ± SD (**p < 0.01, and ***p < 0.001; *n* = 3).

NAC is a potent antioxidant and ROS scavenger. It is able to reduce the excessive lipid peroxidation induced by BCG infection in macrophages through the scavenging of ROS. To test the effect of NAC on BCG-induced macrophage ferroptosis, RAW264.7 cells were preincubated in medium containing 2.0 mM of NAC for 1 h prior to being infected with BCG. As expected, the treatment of NAC significantly increased anti-lipid peroxidation factor Gpx4 in BCG-infected cells in a dose-dependent manner (**Supplementary Figure S5A**). The NAC treatment alone significantly increased the cell viability (**Figure 5A**) and reduced intracellular Fe^2+^ concentration (**Figure 5B**), intracellular ROS production (**Figure 5C**, **Supplementary Figure S5C**), and lipid peroxidation (**Figures 5D, E** and **Supplementary Figure S5C**) of RAW264.7 cells in response to the BCG infection. Molecular analysis further demonstrated that the pretreatment of NAC increased the expression of Gpx4 and Fsp1, but decreased Hmox1 proteins in BCG-infected cells (**Figure 5F**). These data support the notion that intracellular ROS plays a key role in ferroptosis of macrophages in response to Mtb infection (Kuang et al., 2020), and a modulation of *Hmox1* gene may alter ROS production and ferroptosis.

### 3.5 Knockdown *Hmox1* increases macrophage ferroptosis in response to BCG infections

**Figure 6:**
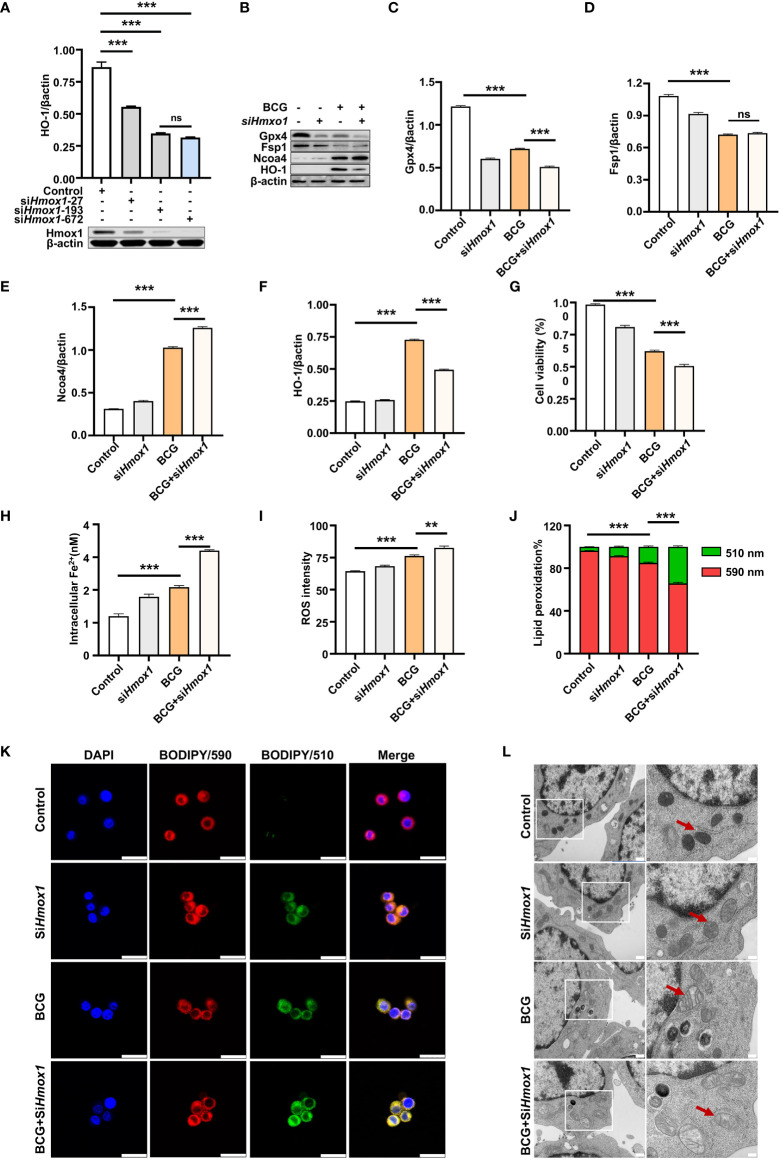
Knockdown of *Hmox1* increases BCG-induced macrophage ferroptosis. **(A)** Representative blots of Hmox1 protein in RAW264.7 cells transfected with siRNA to murine *Hmox1* gene. siRNA was transfected by Lipofectamine™ RNAiMAX, and the protein was analyzed at 24 h post-transfection. The si-*Hmox1* showed the most efficient knockdown of *Hmox1* and was used in subsequent experiments. **(B–E)** Representative blots **(B)** and semi-quantitative analysis of Gpx4 **(C)**, Fsp1 **(D)**, Ncoa4 **(E)**, and HO-1 **(F)** proteins of RAW264.7 cells treated with indicated conditions. The siRNA-mediated knockdown of *Hmox1* amplified the inhibition of Gpx4 and Fsp1 expression in cells infected with BCG. **(G–J)** The viability **(B)**, intracellular Fe^2+^ **(C)**, intracellular ROS **(D)**, and lipid peroxidation **(E)** of RAW264.7 macrophages treated with indicated conditions, as determined by Trypan Blue assay, iron ion probes, flow cytometry, and BODIPY 581/591 C11 assays, respectively. **(K)** Representative fluorescence images of BODIPY 581/591 C11-labeled lipoxidation of polyunsaturated fatty acids in RAW264.7 macrophages of the indicated conditions showed the increase of BCG-induced lipoxidation in cells transfected with siRNA to *Hmox1*. Cell nuclei were counterstained with DAPI. **(L)** Representative images of transmission electron microscopy showed mitochondrial membrane ridge breaks (arrows) in siRNA-transfected RAW264.7 and/or BCG-infected macrophages; the right panel shows the enlarged image of the boxed area in its corresponding image in the left panel. Data obtained from three independent experiments were processed using GraphPad Prism 8.0.1 software and ImageJ 1.52.a. One-way ANOVA was used to analyze the differences between groups. All values are presented as mean ± SD (**p < 0.01; ***p < 0.001; *n* = 3). Bars, 500 nm in the right panel and 200 nm in the left panel of K, and 25 μm in L. ns, no statistical difference.

**Figure 7:**
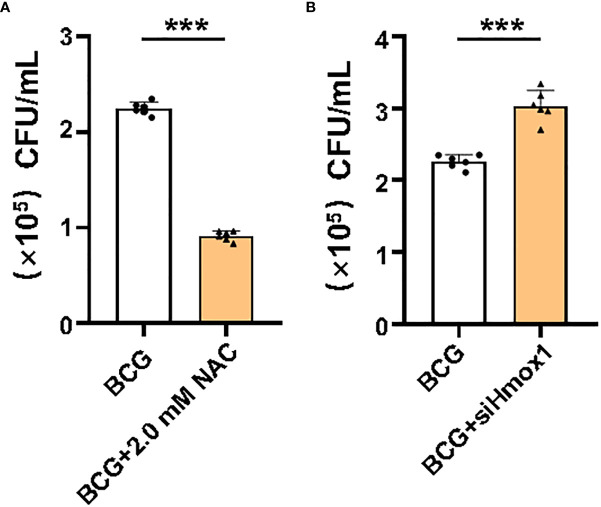
Effect of ROS and Hmox1 on intracellular bacterial loads in RAW264.7 macrophages. Intracellular ROS was scavenged by NAC treatment, and the expression of Hmox1 was inhibited by the transfection of siRNA to *Hmox1*. NAC-pretreated or si*-Hmox1*-transfected RAW264.7 macrophages were incubated with BCG at an MOI of 5 for 1 h, and cells were rinsed to remove uninfected bacteria prior to being cultured with fresh medium for an additional 24 h. The bacteria released in the culture medium was counted by CFU assay. **(A)** The count of colonies in medium of cells pretreated with NAC. **(B)** The count of colonies in medium of cells transfected with si*-Hmox1*. The NAC pretreatment significantly reduced bacteria released from cell necrosis/ferroptosis death, while si***-****Hmox1-*mediated knockdown of *Hmox1* gene strikingly increased CFU count in RAW264.7 cells infected with BCG. Data obtained from three independent experiments were processed using GraphPad Prism 8.0.1 software. Unpaired *t*-test was used to analyze the differential changes of the two groups. ***p < 0.001; n = 3.

HO-1 is a key enzyme against oxidative stress by inhibiting ROS production and reducing ROS cytotoxicity (Seiwert et al., 2020); therefore, an inhibition of Hmox1 may impact the ROS production and ferroptosis in macrophages in response to Mtb infections. To test this hypothesis, the expression of *Hmox1* gene in RAW264.7 cells was knockdown by transfection of siRNA to *Hmox1* (**Figure 6A**). The three siRNA candidates to *Hmxo1* exhibited an ability to knock down the gene expression at the protein level. *siHmxo1*-193 and *siHmxo1*-172 showed more efficiency in the inhibition of HO-1 expression compared to *siHmxo1*-27, but the inhibition mediated by *siHmxo1*-193 and *siHmxo1*-172 showed no difference (**Figure 6A**). The mixture (*siHmxo1*) of equal molar ratio of *siHmxo1*-193 and *siHmxo1*-172 was therefore employed for further experiments in this report. In addition to the reduced expression of HO-1 protein, the siRNA-mediated knockdown of *Hmox1* amplified the inhibition of the expression of Gpx4 and Fsp1, and the induction of Ncoa4 expression in cells infected with BCG (**Figures 6B–F**). Functionally, the siRNA-mediated reduction of Hmox1 significantly amplified the BCG-inhibited cell viability (**Figure 6G**), and BCG-induced intracellular Fe^2+^concentration (**Figure 6H**), intracellular ROS production (**Figure 6I**, **Supplementary Figure S6A**), and lipid peroxidation (**Figures 6J, K** and **Supplementary Figure S6B**) in RAW264.7 cells. The knockdown of *Hmox1* alone also exhibited the ability to inhibit cell viability (**Figure 6G**), and increase intracellular Fe^2+^ concentration (**Figure 6H**) and ROS production (**Figure 6I** and **Supplementary Figure S6A**) and lipid peroxidation (**Figures 6J, K** and **Supplementary Figure S6B**) to some extent in cells uninfected with BCG. In addition, morphological observation revealed worsening mitochondrial membrane ridge breaks in siRNA-transfected RAW264.7, compared to untransfected macrophages infected with BCG (**Figure 6L**). More importantly, the pretreatment of NAC significantly inhibited the release of BCG into the culture medium from lytic death of cells caused by necroptosis/ferroptosis during the infection (**Figure 7A**), while *Hmox1* knockdown induced the BCG release (**Figure 7B**), compared to BCG-infected untreated cells as determined by the CFU assay (**Figures 7A, B**). These results clearly suggest the importance of HO-1-modulated ROS in ferroptosis of macrophages against *Mtb* infections.

## 4 Discussion

Macrophages are the host cells and the frontline defense against Mtb infection, and the form of death of infected macrophages plays a pivotal role in the outcome of Mtb infections. Therefore, a better understanding of the mechanisms of macrophage death induced by Mtb infection will allow us to identify novel targets for HDT in TB treatments. Ferroptosis is a PCD induced by overwhelming lipid peroxidation reactions, and one of mechanisms of Mtb spread following the infection. In the present study, we found that heme oxygenase-1 (*HMOX1*) and pro-ferroptosis cytokines were upregulated, but glutathione peroxidase 4 (*GPX4*) and other key anti-lipid peroxidation factors were downregulated in TB patients and lungs of mice infected with BCG. A mechanistic study further demonstrated that HO-1 regulated the lipid peroxidation-mediated production of ROS and ferroptosis in macrophages in response to BCG infection. These findings suggest that HO-1 is an essential regulator of Mtb-induced ferroptosis, which regulates ROS production and alters macrophage death in response to Mtb infections, suggesting that HO-1 is a potential target for developing HDT in TB treatments.

Metabolic interactions between host cells (macrophages) and pathogen (Mtb) significantly affect the host immune responses and the proliferation of pathogen, and the outcome of Mtb infection (Cambier et al., 2014; Olive and Sassetti, 2016). Iron is one of the most important substances essential to the growth of both pathogen and host cells, which is required for the maintenance of macrophage functions, and the survival of intracellular Mtb (Baatjies et al., 2021). Therefore, the competition in the use of iron (Fe^2+^) source between the macrophages and Mtb may affect the intracellular level of Fe^2+^, and may be critical for the determination of the form of host cell death (necroptosis or apoptosis) and the fate of intracellular pathogens (eliminated or escaped) (Jones and Niederweis, 2011; Mitra et al., 2019; Zhang et al., 2020; Rodriguez et al., 2022).

Ferroptosis is mainly triggered by extra-mitochondrial lipid peroxidation (Dixon et al., 2012), which is characterized by lipid peroxidation as a consequence of iron-dependent accretion of ROS (Dixon and Stockwell, 2014; Amaral et al., 2019). This new form of cell death was also corroborated in macrophages in response to Mtb infection, and it was the main cause of lung tissue necrosis caused by Mtb (Amaral et al., 2019). This finding has gained a great interest in the field of TB research. In the present study, differential expression of genes related to ferroptosis was identified in peripheral blood of TB patients by transcriptome analysis. In particular, the ferroptosis signature gene *GPX4* was downregulated, accompanied by the upregulation of *HMOX1* gene, HO-1, and Hemin content in TB. These observations were further confirmed in RAW264.7 cells infected with BCG. The *in vitro* study demonstrated that HO-1-regulated ROS played a key role in macrophage ferroptosis induced by BCG infection. Mechanistically, scavenging ROS with NAC inhibited BCG-induced macrophage ferroptosis and the release of BCG into the culture medium caused by necroptosis/ferroptosis during the infection, while siRNA-mediated knockdown of *Hmox1* gene expression resulted in opposite effects to NAC treatments. Our results suggest that HO-1 plays a key role in Mtb-induced ferroptosis, through a mechanism by which it regulates ROS production and alters macrophage death in response to Mtb infections.

Heme is a major source of iron for Mtb growth and survival, implying that Mtb has evolved a complex heme enzymatic mechanism (Jones and Niederweis, 2011; McLean and Munro, 2017; Mitra et al., 2019; Zhang et al., 2020). During the Mtb infection, increased ROS production, oxidative mitochondrial damage, and the inducible isoform of HO-1 were observed in macrophages (Amaral et al., 2016; Amaral et al., 2019). All these lines of evidence suggest that ferroptosis may be a primary form of Mtb-induced macrophage death (Amaral et al., 2016; Amaral et al., 2019). Functionally, HO-1 catalyzes the degradation of oxidant heme into biliverdin, iron, and carbon monoxide (CO) (Szade et al., 2021). HO-1 was significantly upregulated in TB patients and in experimental animals infected with Mtb, and was a potential target for HDT for TB (Chinta et al., 2021; Uwimaana et al., 2021). In the present report, the increase of both HO-1 protein and Hemin content, a main source of iron for Mtb and host cells, was also observed in peripheral blood of TB patients, at both transcriptional and/or translational levels, which was strongly correlated with several cytokines. In particular, it was negatively associated with the level of ferroptosis inhibitors (regulators) GPX4 and FSP1 in peripheral blood of TB patients and RAW264.7 cells infected with BCG.

Of great interest, the BCG-inhibited Gpx4 was only observed in RAW264.7 cells infected with BCG at a low dose (MOI = 5). A high dose of BCG infection (MOI of 10 and 15) induced Gpx4 expression in this type of cells (**Figures 2D, E**). Flow cytometry assay further revealed that the low dose (MOI = 5) of BCG infection induced necrotic cell death, while the high doses (MOI = 10 and 15) of BCG promoted apoptotic cell death in RAW264.7 cells (**Figure 2J**). These data suggest that a low dose of Mtb infection favors macrophage ferroptosis, but an increased load of bacteria in a certain range may induce cell apoptosis. We currently do not fully understand the underlying mechanism why the dynamic changes of Gpx4 in RAW264.7 cells infected with different doses of BCG, i.e. an inhibition of Gpx4 expression at an MOI of 5, while an induction at an MOI of 10 and 15.

Biochemistry studies demonstrate that ferroptosis is mainly induced through lipid ROS generated by the iron-catalyzed Fenton reaction. Fenton reaction requires ROS and ferrous iron for initiation and space of the integrity of cells (Tang et al., 2021) (Stockwell, 2022). Indeed, scavenging ROS with NAC significantly increased Gpx4, and reduced ferroptosis and intracellular bacteria release in RAW264.7 cells in response to BCG infection. In addition, cell ferroptosis does not produce apoptotic vesicles as cell apoptosis does, and does not cause severe cell morphological changes as necrosis either; therefore, it maintains the cell integrity and favors Fenton reaction and leads the infected RAW264.7 cells to undergo ferroptosis at an MOI of 5 in this study. In contrast, the increase of bacteria load with an MOI of 10 or 15 in RAW264.7 cells induced cell apoptosis to remove the number of intracellular bacteria (**Figure 2J**), which might in turn inhibit cell ferroptosis by two possible mechanisms. First, the process of formation of apoptotic vesicle has the potential to reduce intracellular ROS and block Fenton reaction. Second, divalent iron is highly oxidizing and easily converted into trivalent iron due to loss of electrons (Sukhbaatar and Weichhart, 2018; Nairz and Weiss, 2020). Trivalent iron is stored in ferritin of cells, while only cells in a viable state have an intact iron metabolism able to degrade ferritin, subsequentially release trivalent iron from ferritin, and ultimately reduce the trivalent iron to divalent iron (Li et al., 2020; Mesquita et al., 2020). However, whether the increase of apoptosis is correlated with a reduced ferroptosis needs further study.

ROS generated from lipid peroxidation is crucial in cell ferroptosis (Conrad et al., 2018), and HO-1 is an essential cytoprotective enzyme that inhibits inflammation and oxidative stress (Araujo et al., 2012; Rockwood et al., 2017; Seiwert et al., 2020; Chinta et al., 2021; de Oliveira et al., 2022), and a target for HDT of TB (McLean and Munro, 2017; Chinta et al., 2021). The expression of HO-1 is largely dependent on oxidative stress in Mtb-infected cells, where an elevated expression of HO-1 is a strategy of host cells in response to oxidative stress triggered by intracellular bacteria (Rockwood et al., 2017). HO-1 exhibits an ability to inhibit ROS formation, DNA damage, and cytotoxicity induced by heme iron in human colonocytes (Seiwert et al., 2020). Importantly, the interplay between HO-1 and iron metabolism has been demonstrated to play a critical role in modulating immune responses of macrophages, as iron is a key product of HO-1 activity for cellular biological processes in both eukaryotic cells and bacteria (Li and Stocker, 2009). Therefore, HO-1 regulates intracellular iron levels to modulate the cellular oxidation and immune responses of macrophages (de Oliveira et al., 2022). Despite the fact that HO-1 is known to play a protective role in host cells during an infectious process, the upregulated HO-1 in Mtb-infected cells increases intracellular iron level and ROS production, and subsequentially leads to lipid peroxidation and ferroptosis (Yang et al., 2022). In this report, the siRNA-mediated reduction of *Hmox1* expression increased ROS production, lipid peroxidation, and intracellular iron, and further induced ferroptosis in RAW264.7 cells infected with BCG at an MOI of 5. In addition, the siRNA silence of *Hmox1* gene also increased expression of iron autophagy protein Ncoa4, a critical cytokine in the maintenance of iron homeostasis (Bellelli et al., 2016). Together, the findings of others and this study, and the discrepancy of results from different studies imply the complicated and dynamic biological process of ferroptosis in cells in response to Mtb infections. The process of ferroptosis is tightly regulated, and may depend on the species, dose and virulence of pathogen, and host cell-type context, which requires further investigation.

Collectively, in the present report, we revealed the upregulation of *HMOX1* but a downregulation of *GPX4* in TB patients, and lungs of mice infected with BCG. An *in vitro* mechanistic study using murine macrophage-like RAW264.7 cells further demonstrated that Hmox1 regulated intracellular levels of ROS and Fe^2+^, and subsequentially modulated cell ferroptosis induced by BCG infection. The siRNA-mediated knockdown of *Hmox1* gene increased intracellular ROS, Fe^2+^, and Ncoa4, and promoted cell ferroptosis and the release of intracellular BCG, while scavenging ROS demonstrated opposite effects to that of siRNA-mediated knockdown of *Hmox1* gene. Our results suggest that HO-1 is an essential regulator of Mtb-induced ferroptosis, which regulates ferroptosis by modulating intracellular ROS production and Fe^2+^ to alter macrophage death against Mtb infections.

## Data availability statement

The datasets presented in this study can be found in online repositories. The names of the repository/repositories and accession number(s) can be found in the article/supplementary material.

## Ethics statement

This study was reviewed and approved by The Ethic Committee of Human Research at General Hospital of Ningxia Medical University. The patients/participants provided their written informed consent to participate in this study. The animal study was reviewed and approved by The Laboratory Animal Welfare Ethics Review Committee of Ningxia University.

## Author contributions

CM: conception, acquisition, analysis, interpretation, writing, revision, and editing. XW: acquisition and analysis. XZ: acquisition and analysis. XL: conception, acquisition, analysis, interpretation, writing, revision, editing, and overall supervision. GD: conception, analysis, interpretation, writing, revision, editing, and overall supervision. All authors contributed to the article and approved the submitted version.

## Funding

This work was supported by a grant from the National Natural Science Foundation of China (No. 32160162).

## Acknowledgments

The authors thank Mr. Fuyang Song, Dr. Jing Zeng, Dr. Zhanbing Ma, and Dr. Xue Lin for their technical and bioinformatic assistances. We also thank Miss Jia Ma, Mr. Jialin Yu, and Miss Zhaoqian Gong for valuable discussions.

## Conflict of interest

The authors declare that the research was conducted in the absence of any commercial or financial relationships that could be construed as a potential conflict of interest.

## Publisher’s note

All claims expressed in this article are solely those of the authors and do not necessarily represent those of their affiliated organizations, or those of the publisher, the editors and the reviewers. Any product that may be evaluated in this article, or claim that may be made by its manufacturer, is not guaranteed or endorsed by the publisher.
