# Corrigendum: Heme oxygenase-1 modulates ferroptosis by fine-tuning levels of intracellular iron and reactive oxygen species of macrophages in response to *Bacillus Calmette-Guerin* infection

**DOI:** 10.3389/fcimb.2022.1105336

**Published:** 2022-12-16

**Authors:** Chenjie Ma, Xiaoling Wu, Xu Zhang, Xiaoming Liu, Guangcun Deng

**Affiliations:** ^1^ Key Laboratory of Ministry of Education for Conservation and Utilization of Special Biological Resources in the Western China, Ningxia University, Yinchuan, China; ^2^ School of Life Science, Ningxia University, Yinchuan, China; ^3^ Department of Beijing National Biochip Research Center sub-center in Ningxia, General Hospital of Ningxia Medical University, Yinchuan, China; ^4^ Department of Anatomy and Cell Biology, University of Iowa, Carver College of Medicine, Iowa City, IA, United States; ^5^ Analysis and Testing Center, Ningxia University, Yinchuan, China

**Keywords:** Heme oxygenase-1, ferroptosis, macrophage, *Mycobacterium tuberculosis*, Bacillus Calmat and Guerin

## Error in figure/table

In the original article, there were several mistakes in **Figure 2H**; **Figure 2I**; **Figure 3B**; **Figure 5F** as published. Some errors in Figures were introduced during the organization of images in some figures: (1) The histogram plots in **Figure 2H** and **Figure 2I** were incorrectly exchanged. (2) **Figure 3B** and **Figure 6L** showed duplicate electron micrographs. The image in **Figure 6L** was correct, while the image in **Figure 3B** was incorrect and need to be replaced. (3) **Figure 5F** and **Figure 6B** showed duplicate images of β-actin blots. The β-actin blot in **Figure 6B** was correct, while the image in **Figure 5F** was incorrect and need to be replaced.

**Figure 2:**
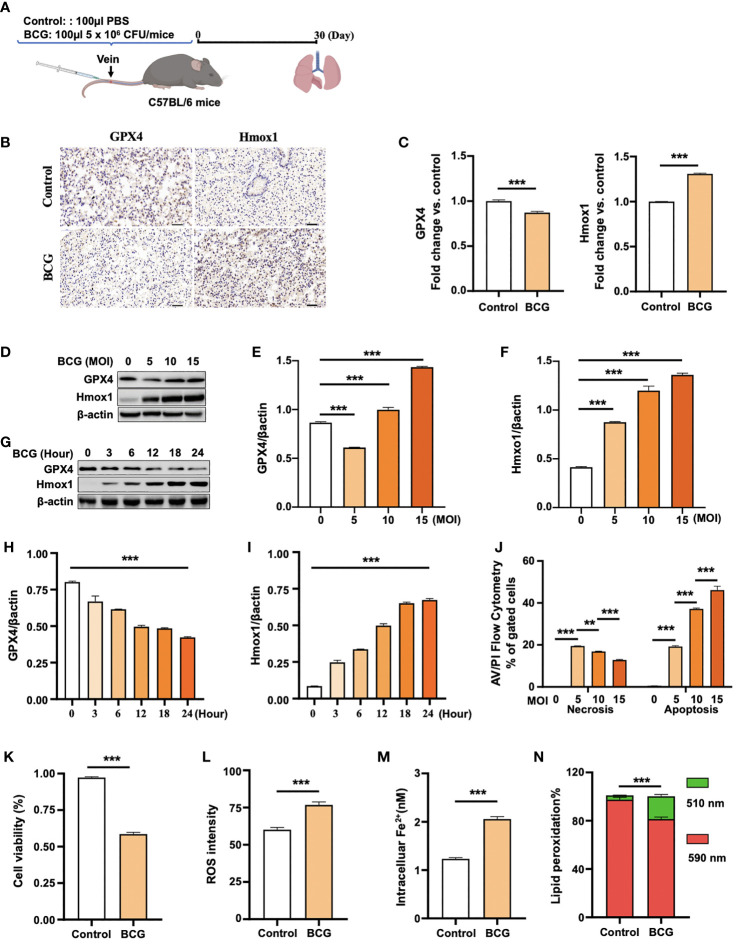
BCG induces ferroptosis in macrophages. **(A)** Schematic diagram shows the infection of mice with BCG *via* tail vein injection. The infected mice are analyzed at 30 days post-infection (DPI). **(B, C)** C57BL/6 mice were injected with 5 × 10^6^ CFU BCG in 100 µl *via* the tail vein and the lungs were harvested for evaluating the expression of Gpx4 and HO-1 proteins at 30 DPI by immunohistochemical (IHC) assay **(B)** and semi-quantified by ImageJ-IHC Profiler **(C)**. Lungs of mice infected with BCG showed less and more abundant Gpx4 and HO-1 proteins compared to the uninfected controls, respectively. Bar represents 500 μm in **(B)** **(D-F)** RAW264.7 murine macrophage-like cells were infected with BCG at indicated MOI for 24 h, and the abundance of Gpx4 and HO-1 proteins was examined by Western blotting assay. The representative blots **(D)** and semi-quantification of Gpx4 **(E)** and HO-1 **(F)** showed a dose-dependent induction of Gpx4 and HO-1 in this type of cells, except an inhibition of Gpx4 expression in cells infected with a low dose of BCG at an MOI of 5. **(G–I)** The representative blots **(D)** and semi-quantification of Gpx4 **(H)** and HO-1 **(I)** demonstrated a time-dependent inhibition of Gpx4 and induction of HO-1 in RAW264.7 infected with BCG at an MOI of 5 for a 24-h time period. **(J)** Induction of cell necrosis and apoptosis in RAW264.7 cells at 24 h post-infection of BCG at the indicated MOI determined by using Annexin V/PI in flow cytometry. **(K)** Cell viability assay showed that the infection of BCG decreased the viability of RAW264.7 cells at an MOI of 5 for 24 has detected by Trypan Blue assay. **(L)** The infection of BCG induced the production of intracellular ROS in RAW264.7 cells at an MOI of 5 for 24 has detected by flow cytometry assay. **(M)** The infection of BCG increased the concentration of intracellular Fe^2+^ in RAW264.7 cells at an MOI of 5 for 24 has detected using iron ion probes. **(N)** The infection of BCG induced lipid peroxidation in RAW264.7 cells at an MOI of 5 for 24 h as determined by BODIPY 581/591 C11 assays. Upon oxidation, its excitation of Red/590 nm shifts to 510 nm (Green). The ratio of Green/Red cells in the BCG-infected cells was 17.22%, while the uninfected cells was 3.52%. Data obtained from three independent experiments were processed using GraphPad Prism 8.0.1 software and ImageJ 1.52.a. Unpaired *t*-test was used to analyze the differential changes of the two groups. One-way ANOVA and Tukey’s multiple comparisons test was used to analyze the differential changes of multiple groups. Data represented mean ± SD from three independent experiments; significant differences are indicated with asterisks (**p < 0.01; ***p < 0.001).

**Figure 3:**
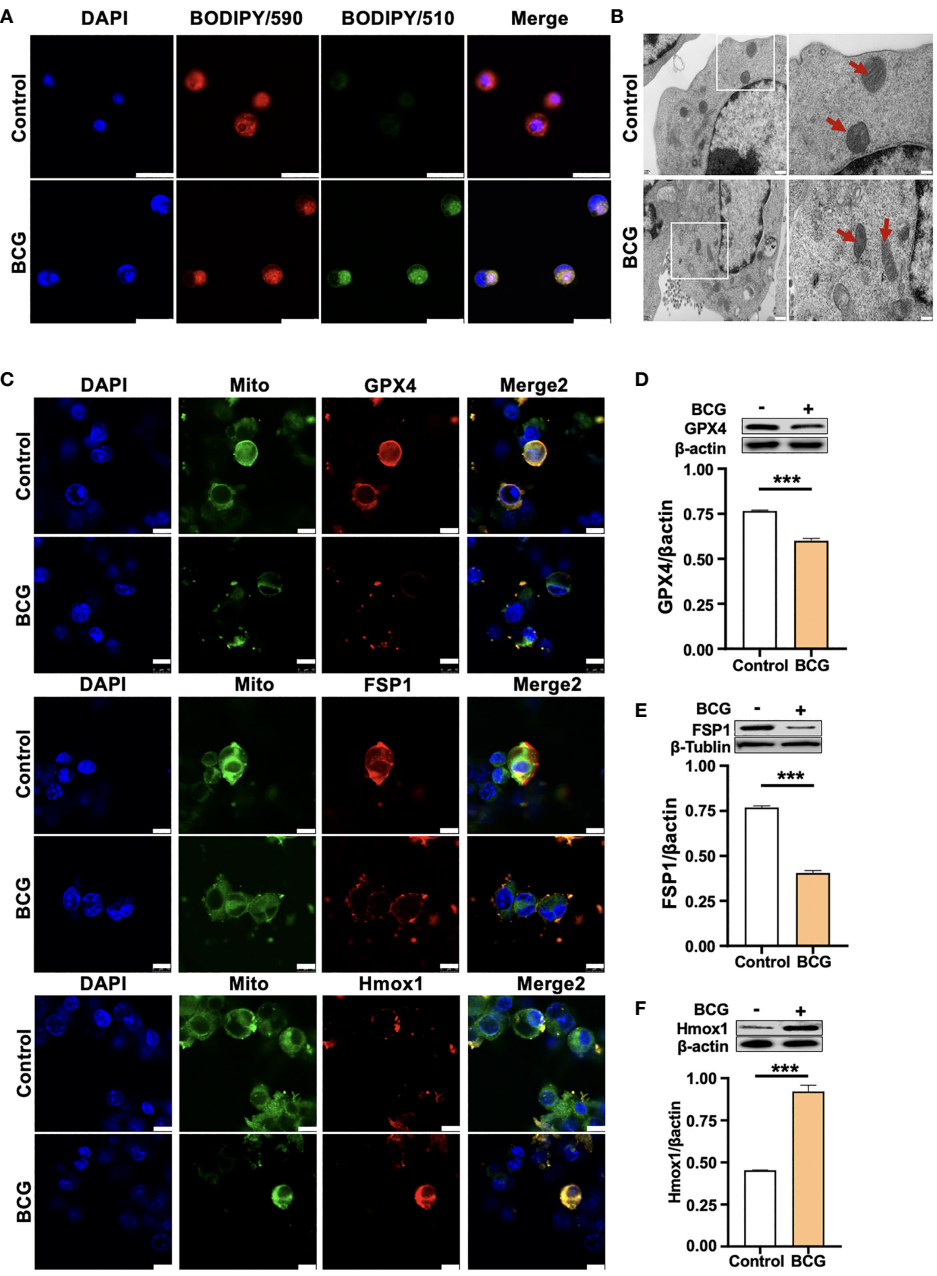
HO-1 is involved in the BCG-induced macrophage ferroptosis. **(A)** Representative images of BODIPY 581/591 C11-labeled lipoxidation of polyunsaturated fatty acids. BCG-infected RAW264.7 cells showed a strong positive lipid peroxidation (Green/510 nm) compared to the uninfected cells. **(B)** Representative images of transmission electron microscopy showed mitochondrial membrane ridge breaks (arrows) in RAW264.7 macrophages infected by BCG; the right panel shows the enlarged image of the boxed area in its corresponding image in the left panel. **(C)** Representative immunofluorescence images of Gpx4 (top panels), Fsp1 (middle panels), and HO-1 (bottom panels) showed the decrease of anti-ferroptotic markers Gpx4 and Fsp1, but increased pro-ferroptotic marker HO-1 in BCG-infected cells. Cell mitochondria were labeled with CellLight™ Mitochondria-GFP (green), which were reduced in macrophages following the BCG infection. **(D–F)** Representative blots and semi-quantitative analysis of Gpx4, Fsp1, and HO-1 proteins of RAW264.7 cells. Statistical analysis of data performed using GraphPad Prism 8.0.1 software and ImageJ 1.52.a. Cell nuclei were counterstained with DAPI. Data obtained from three independent experiments were processed using GraphPad Prism 8.0.1 software and ImageJ 1.52.a. Unpaired *t*-test was used to analyze the differential changes of two groups. Data are presented as mean ± SD from three independent experiments (***p < 0.001; *n* = 3). Bars, 500 nm in the right panel and 200 nm in the left panel of A, 25 μm in B, and 10 μm in C.

**Figure 5:**
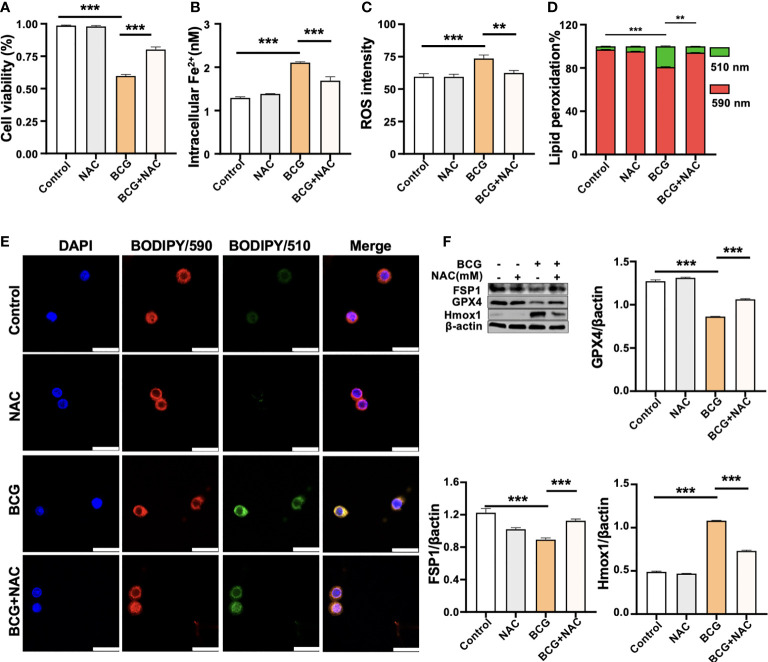
ROS scavenger NAC reduces BCG-induced macrophage ferroptosis. RAW264.7 macrophages were preincubated in medium containing 2.0 mM NAC for 1 h prior to being infected with BCG at an MOI of 5 for 24 h, before they were harvested for analysis. **(A–D)** The viability **(A)**, intracellular Fe^2+^ **(B)**, intracellular ROS **(C)**, and lipid peroxidation **(D)** of RAW264.7 macrophages treated with the indicated conditions, as determined by Trypan Blue assay, iron ion probes, flow cytometry, and BODIPY 581/591 C11 assays, respectively. **(E)** Representative fluorescence images of BODIPY 581/591 C11-labeled lipoxidation of polyunsaturated fatty acids in RAW264.7 macrophages of the indicated conditions showed the reduction of BCG-induced lipoxidation in cells pretreated with NAC. Cell nuclei were counterstained with DAPI. **(F)** Representative blots and semi-quantitative analysis of Gpx4, Fsp1, and HO-1 proteins of RAW264.7 cells treated with the indicated conditions. The NAC pretreatment increased Gpx4 and Fsp1 expression, but decreased Hmox1 protein in BCG-infected cells. Data obtained from three independent experiments were processed using GraphPad Prism 8.0.1 software and ImageJ 1.52.a. All values are presented as mean ± SD (**p < 0.01, and ***p < 0.001; *n* = 3).

## Figure correction

In the original article, there were four typographical errors. Corrections have been made to **Figures**. (1) BCG induces ferroptosis in macrophages; (2) HO-1 is involved in the BCG-induced macrophage ferroptosis; (3) ROS scavenger NAC reduces BCG-induced macrophage ferroptosis. (1) 08 (2) 09 (3)11:

(1) The histogram plots in **Figure 2H** and **Figure 2I** were exchanged. The expression of gpx4 showed in **Figure 2H** was gradually decreased, while the expression of Hmox1 showed in **Figure 2I** gradually increased.
(2) Replaced the image in **Figure 3B** file with the correct image of electron microscopy.
(3) The original image of β-actin in **Figure 5F** was replaced.

The authors apologize for these errors and state that this does not change the scientific conclusions of the article in any way. The original article has been updated.

