# Supplementary material for: Heme oxygenase-1 modulates ferroptosis by fine-tuning levels of intracellular iron and reactive oxygen species of macrophages in response to Bacillus Calmette-Guerin infection
Source: Front Cell Infect Microbiol. 2022 Sep 23;12:1004148. doi: 10.3389/fcimb.2022.1004148 (PMC9539760; doi:10.3389/fcimb.2022.1004148)
Supplement: Supplementary file 1 [file DataSheet_1.pdf]

## Supplementary Material

## 1 Supplementary Figures

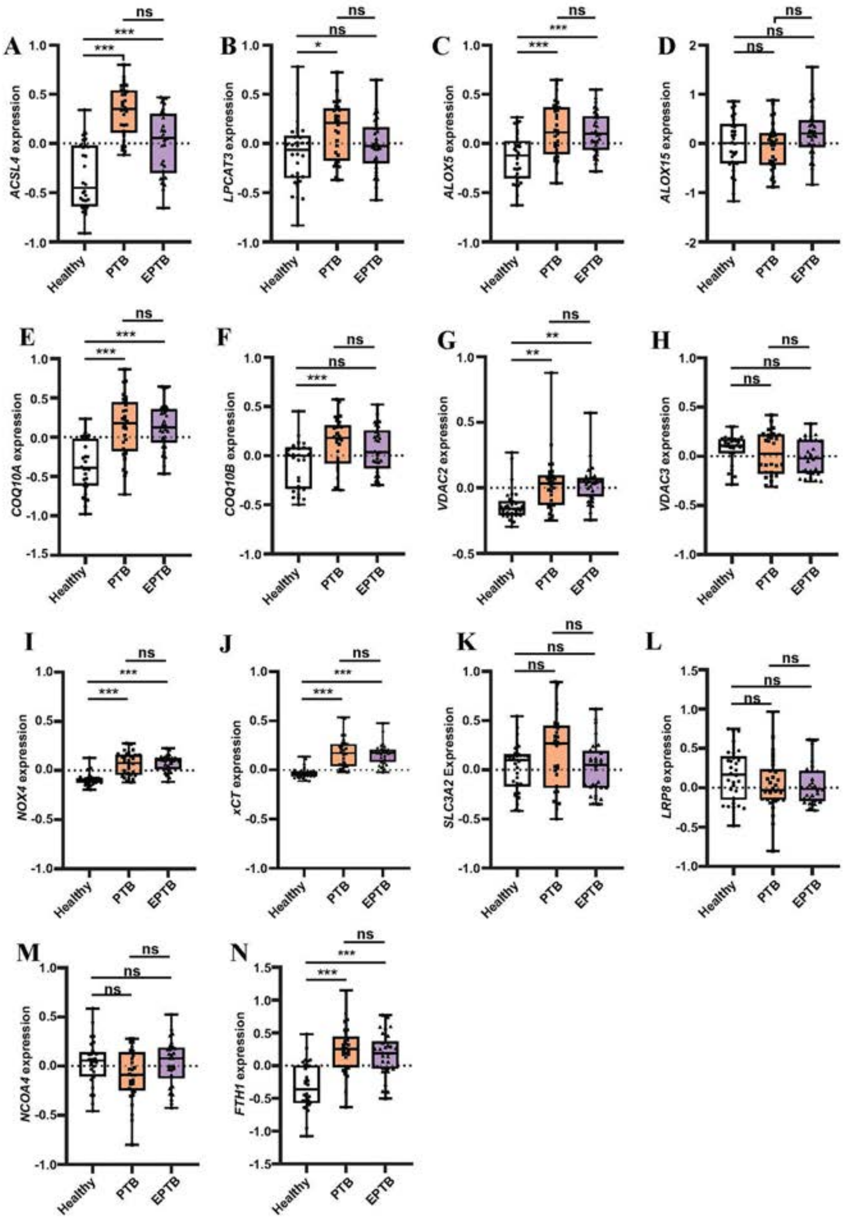


**Supplementary Figure S1. Differentiate expression of genes involved in ferroptosis in peripheral blood of TB patients.** Peripheral blood from healthy individuals (N=30), patients with pulmonary TB (PTB, N=30), and patients with extra-pulmonary TB (EPTB, N=30) were collected for transcriptome analysis using GEO database GSE83456 (a transcriptome sequencing dataset of peripheral blood from TB patients). (A-O) The differentiate expression of transcript of the *ACSL4* (A), *LPCAT3* (B), *ALOX5*

(C), *ALOX15* (D), *COQ10A* (E), *COQ10B* (F), *VDAC2* (G), *VDAC3* (H), *NOX4* (I), *xCT* (J), *SLC3A2* (K), *TFRC* (L), *NCOA4* (M), *FTH1* (N), and *LRP8* (O) in the peripheral blood of TB patients. An increased expression of transcripts of *ACSL4*, *LPCAT3*, *ALOX5*, *COQ10A*, *VDAC2*, *NOX4*, *xCT* and *FTH1* genes was found in peripheral blood of both patients with PTB and EPTB, while an increased *LPCAT3* and *COQ10B* transcripts were only observed in PTB patients, as compared with the healthy individuals. *TFRC* transcript was decreased in peripheral blood of EPTB patients compared with the healthy individuals. There was no statistical significance in differentiate expression of transcripts *ALOX15*, *VDAC3*, *SLC3A2*, *NCOA4* and *LRP8* genes. Data obtained from three independent experiments was processed using GraphPad Prism 8.0.1 software and ImageJ 1.52.a. Unpaired t test was used to analyze the differential changes of the two groups. Data represented mean ± SD, significant differences were indicated with asterisks (**, P＜0.01； ***, P＜0.001) .

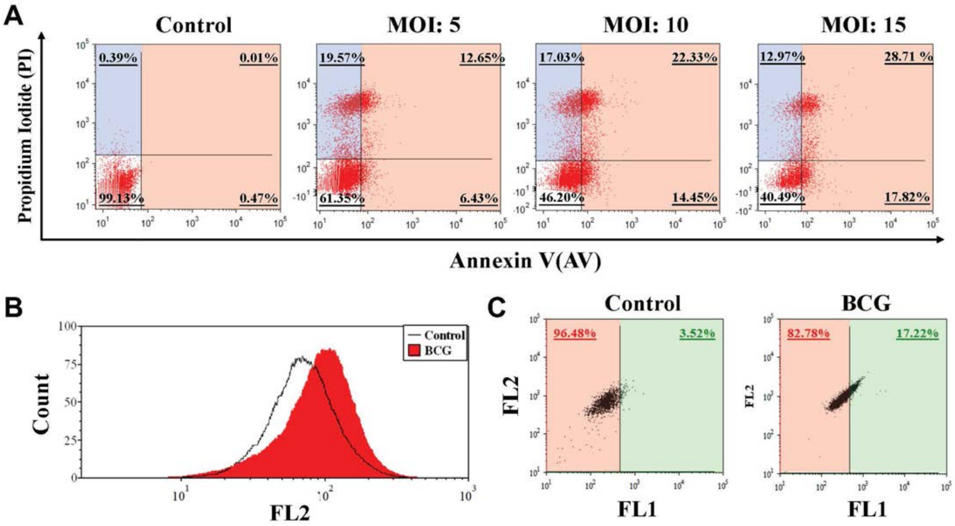


**Supplementary Figure S2. BCG induces necrosis and apoptosis in macrophages.** (A) Representative quadrant plots of cell necrosis and apoptosis of RAW264,7 cells at 24 h post the infection of BCG at MOI of 5, 10 and 15, as determined by using Annexin V/PI in flow cytometry. (B)Histogram represented ROS level produced by treated RAW264.7 cells at MOI of 5 for 24 hours in flow cytometry assay. (C) The infection of BCG induced lipid peroxidation in RAW264.7 cells at MOI of 5 for 24 h as determined by BODIPY 581/591 C11 assays. Upon oxidation, its excitation of Red/590 nm shifts to 510nm (Green). The ratio of Green/Red cells in the BCG-infected cells was 17.22%, while the uninfected cells was 3.52%. One-way ANOVA was used to analyze the differences between groups.

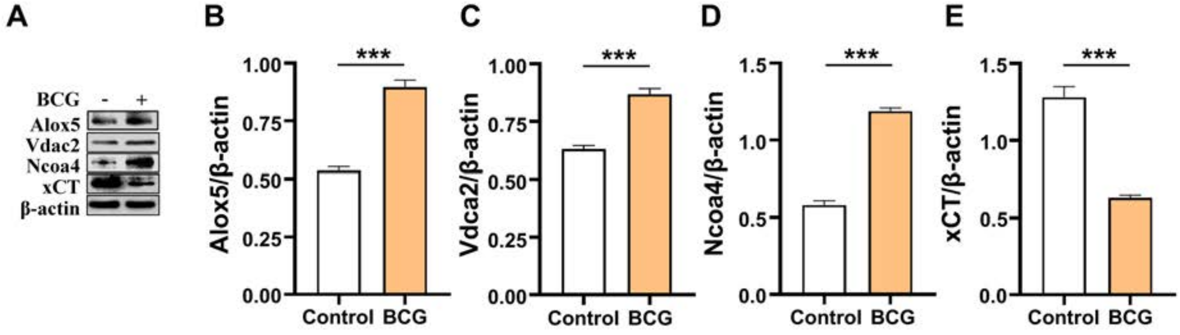


**Supplementary Figure S3. BCG-induced macrophage ferroptosis.** RAW264.7 cells were infected with BCG at MOI of 5 for 24 h prior to being harvest for immunoblotting analysis. (A) Representative blots of immunoblotting assay for indicated proteins of RAW264.7 cells. (B-E) Semi-quantitative analysis of blots of Alox5 (B), Vdca2 (C), Ncoa4 (D) and xCT (E) proteins of RAW264.7 cells showed in A. Data obtained from three independent experiments was processed using GraphPad Prism 8.0.1 software and ImageJ 1.52.a. Unpaired t test was used to analyze the differential changes of the two groups. Data represented as mean ± SD from three independent experiments (***, $p < 0.001$; $n = 3$).

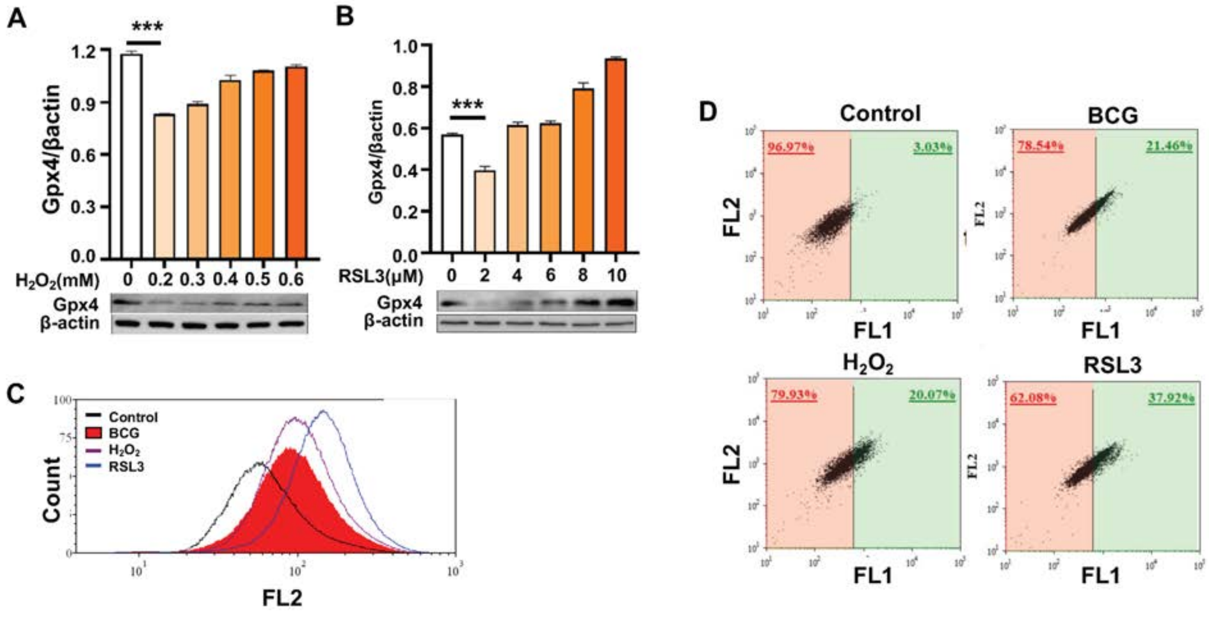


**Supplementary Figure S4. $H_2O_2$ induces ferroptosis in RAW264.7 macrophages.** RAW264.7 macrophages were treated with different concentrations of $H_2O_2$ or RSL3 to determine the optimal concentration that induce cell ferroptosis accessed by the inhibition of Gpx4 expression and flow cytometry. (A) Representative blots of Gpx4 of RAW264.7 cells treated with $H_2O_2$ at concentrations of 0, 0.2, 0.3, 0.4, 0.5 and 0.6 mM. The most inhibitory expression of Gpx4 was observed in cells treated with 0.2 mM of $H_2O_2$. (A) Representative blots of Gpx4 of RAW264.7 cells treated with $H_2O_2$

at concentrations of 0, 2, 4, 6, 8 and 10 mM. The most inhibitory expression of GPX4 was observed in cells treated with 2 mM of RSL3. (C-D) Histogram represented ROS level produced by treated RAW264.7 cells in flow cytometry assay (C) and Dot plot represented lipid peroxidation (D) of RAW264.7 macrophages in response to BCG infection (MOI 5) (top right), $H_2O_2$ (0.2 mM) (bottom left) and ferroptosis agonist RSL3 (2.0 μM) (bottom right), a major participant of the Fenton response at 24 h post treatments, as determined by flow cytometry and BODIPY 581/591 C11 assays respectively. Data obtained from three independent experiments was processed using GraphPad Prism 8.0.1 software and ImageJ 1.52.a. One-way ANOVA and Tukey's multiple comparisons test was used to analyze the differential changes of the three groups. Data represented mean ± SD from three independent experiments, significant differences are indicated with asterisks (***, $P < 0.001$).

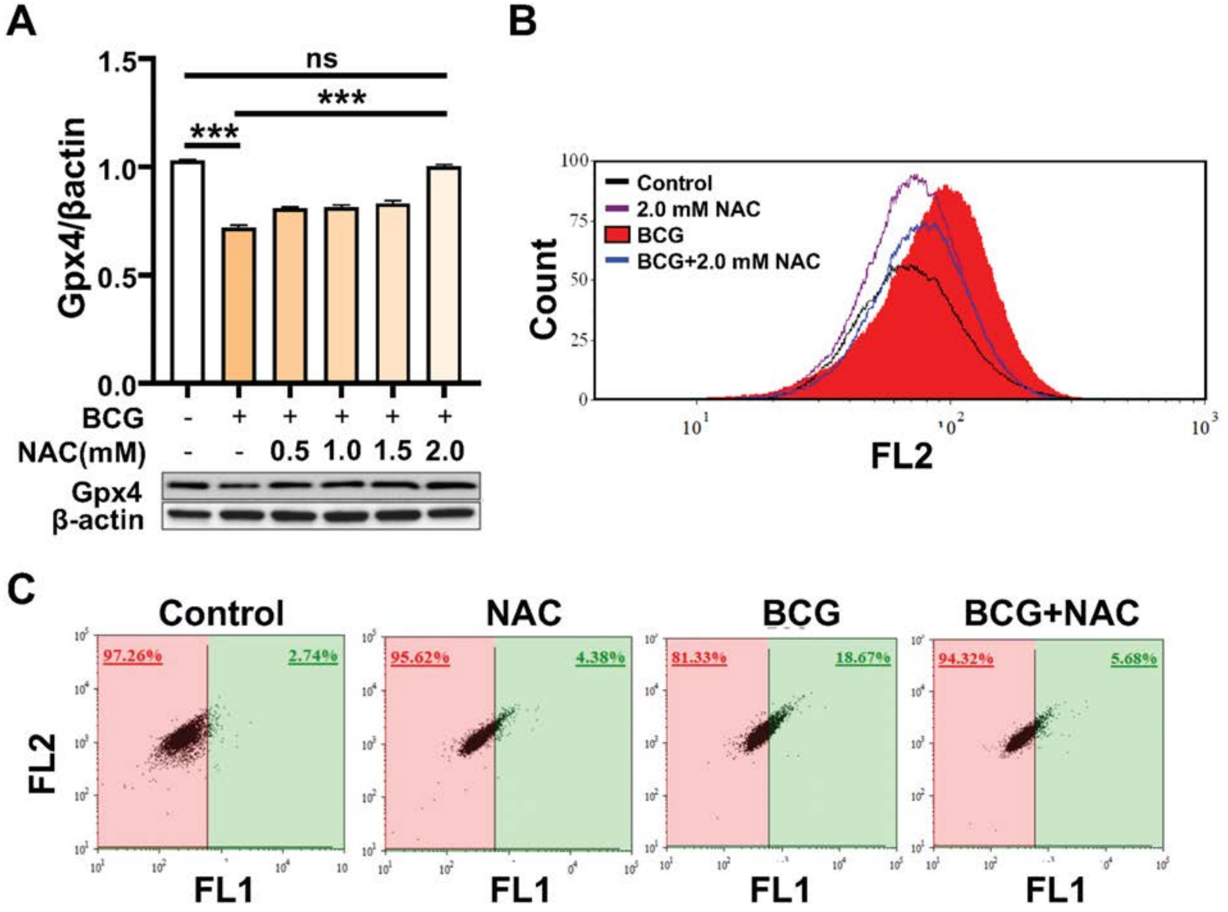


**Supplementary Figure S5. ROS scavenger NAC reduces BCG-induced macrophage ferroptosis.** RAW264.7 macrophages were preincubated in medium containing NAC at a concentration of 0.5, 1.0, 1.5 or 2.0mM for 1 h prior to being infected with BCG at MOI of 5 for 24 hours. (A) Representative blots (bottom panel) and semi-quantitative analysis (top panel graph) of Gpx4 proteins of RAW264.7

cells showed that NAC was able to recover the BCG-inhibited expression of Gpx4 in a dose-dependent manner. (B-C) Histogram represented ROS level (B) and lipid peroxidation (C) of BCG-infected RAW264.7 macrophages that were pretreated with 2.0 mM of NAC, as determined by flow cytometry and BODIPY 581/591 C11 assays respectively. Data obtained from three independent experiments was processed using GraphPad Prism 8.0.1 software and ImageJ 1.52.a. One-way ANOVA and Tukey's multiple comparisons test was used to analyze the differential changes of the three groups. Data represented mean ± SD from three independent experiments, significant differences are indicated with asterisks (***, P < 0.001).

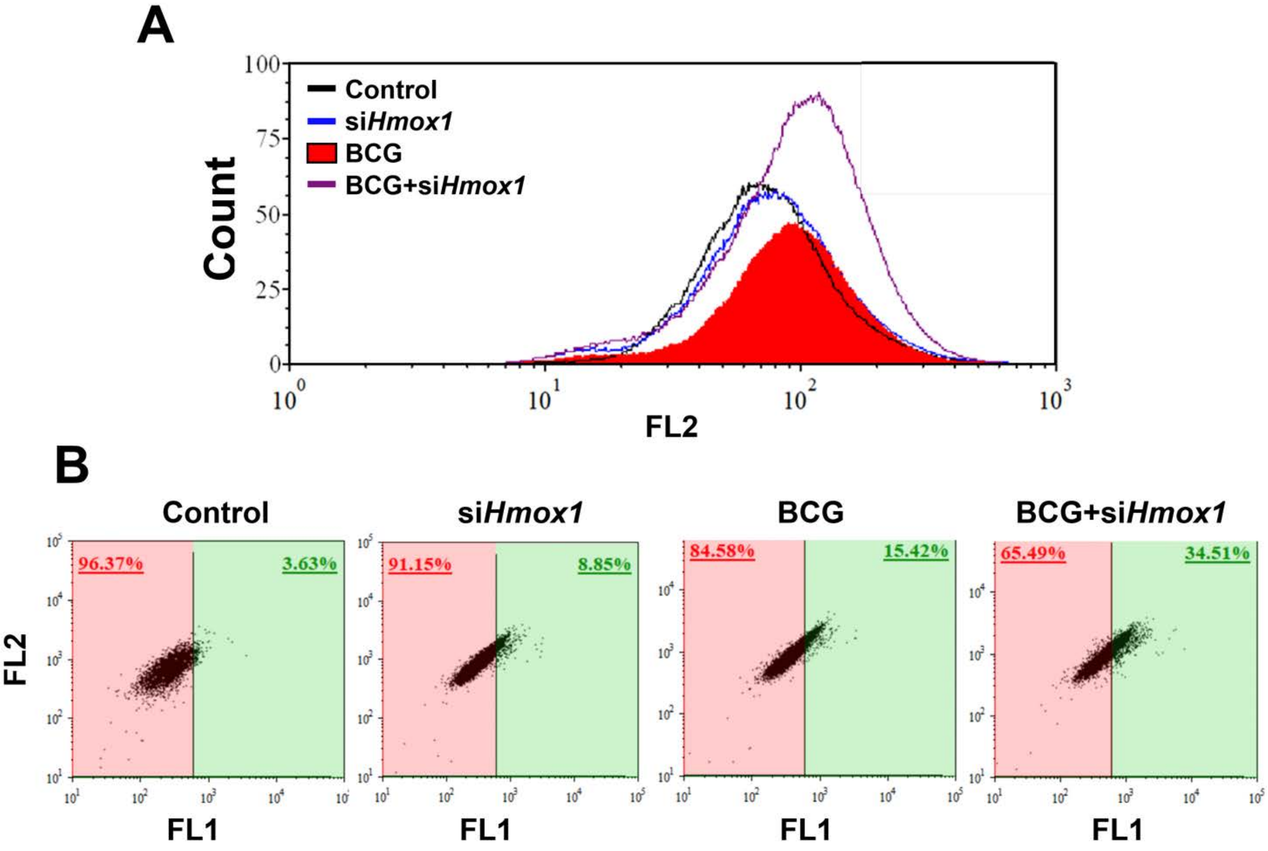


**Supplementary Figure S6. Knockdown of *Hmox1* increases BCG-induced macrophage ferroptosis.** RAW264.7 cells were transfected with si*Hmox1* to murine *Hmox1* gene, followed by being infected with BCG, prior to accessing the intracellular ROS production and lipid peroxidation by cytometry analysis at 24 h post infection. (A-B) Histogram represented ROS level (A) and Dot plot represented lipid peroxidation (B) of BCG-infected RAW264.7 macrophages that were transfected with si*Hmox1*, as determined by flow cytometry and BODIPY 581/591 C11 assays respectively.

## 2 Supplementary Tables

### Supplementary Table S1: List of antibodies used

| Antibody | Host | Reactivity | Mw (Kd) | Vendor | Cat. # |
|---|---|---|---|---|---|
| GPX4 | Rabbit | mouse | 17 | Abclonal | A11243 |
| FSP1 | Rabbit | mouse | 41 | Proteintech | 20886-1-AP |
| Hmox1 | Rabbit | mouse | 28-33 | Proteintech | 10701-1-AP |
| ALOX5 | Rabbit | mouse | 65 | Abclonal | A6864 |
| VDAC2 | Rabbit | mouse | 32 | Proteintech | 11663-1-AP |
| NCOA4 | Rabbit | mouse | 70 | Abclonal | A5695 |
| xCT | Rabbit | mouse | 40-45 | Proteintech | 26864-1-AP |
| β-actin | Rabbit | mouse | 42 | Proteintech | 20536-1-AP |
| β-Tublin | Mouse | mouse | 50 | Abmart | M30109 |
| HRP-conjugated Affinipure Goat Anti-Rabbit IgG(H+L) | Goat | rabbit | NA | Proteintech | SA00001-2 |
| Anti-rabbit IgG Fab2 Alexa Fluor（R）555 Molecular | Goat | rabbit | NA | Cell Signaling | 4413S |

**Supplementary Table S2:** Demographics of individuals of transcriptome analysis

| Group | Sample ID | gender | ethnicity | age | disease state | Source name |
|---|---|---|---|---|---|---|
| Healthy | GSM2203617 | M | Black | 35 | HC | Blood |
| Healthy | GSM2203604 | F | Black | 28 | HC | Blood |
| Healthy | GSM2203598 | M | Black | 49 | HC | Blood |
| Healthy | GSM2203619 | F | White | 22 | HC | Blood |
| Healthy | GSM2203624 | M | White | 34 | HC | Blood |
| Healthy | GSM2203629 | M | ISC | 28 | HC | Blood |
| Healthy | GSM2203603 | F | White | 52 | HC | Blood |
| Healthy | GSM2203640 | F | Other | 20 | HC | Blood |
| Healthy | GSM2203633 | M | White | 54 | HC | Blood |
| Healthy | GSM2203618 | F | ISC | 35 | HC | Blood |
| Healthy | GSM2203608 | F | Black | 46 | HC | Blood |
| Healthy | GSM2203599 | M | White | 23 | HC | Blood |
| Healthy | GSM2203630 | M | Black | 24 | HC | Blood |
| Healthy | GSM2203605 | F | Black | 36 | HC | Blood |
| Healthy | GSM2203597 | F | Black | 47 | HC | Blood |
| Healthy | GSM2203638 | M | ISC | 29 | HC | Blood |

| | | | | | | |
|---|---|---|---|---|---|---|
| Healthy | GSM2203639 | F | White | 68 | HC | Blood |
| Healthy | GSM2203616 | M | Black | 64 | HC | Blood |
| Healthy | GSM2203622 | M | White | 38 | HC | Blood |
| Healthy | GSM2203628 | F | ISC | 23 | HC | Blood |
| Healthy | GSM2203643 | M | ISC | 45 | HC | Blood |
| Healthy | GSM2203625 | M | White | 42 | HC | Blood |
| Healthy | GSM2203642 | M | Black | 32 | HC | Blood |
| Healthy | GSM2203600 | F | Other | 49 | HC | Blood |
| Healthy | GSM2203627 | F | White | 33 | HC | Blood |
| Healthy | GSM2203602 | M | White | 35 | HC | Blood |
| Healthy | GSM2203613 | M | White | 30 | HC | Blood |
| Healthy | GSM2203626 | F | ISC | 34 | HC | Blood |
| Healthy | GSM2203641 | M | White | 31 | HC | Blood |
| Healthy | GSM2203647 | F | White | 29 | HC | Blood |
| PTB | GSM2203663 | F | Other | 34 | PTB | Blood |
| PTB | GSM2203671 | F | Other | 34 | PTB | Blood |
| PTB | GSM2203656 | F | ISC | 27 | PTB | Blood |
| PTB | GSM2203692 | F | White | 25 | PTB | Blood |
| PTB | GSM2203666 | M | Other | 41 | PTB | Blood |
| PTB | GSM2203685 | M | White | 32 | PTB | Blood |
| PTB | GSM2203650 | M | White | 62 | PTB | Blood |
| PTB | GSM2203659 | F | Black | 31 | PTB | Blood |
| PTB | GSM2203689 | M | White | 40 | PTB | Blood |
| PTB | GSM2203677 | M | Other | 25 | PTB | Blood |
| PTB | GSM2203653 | M | ISC | 22 | PTB | Blood |
| PTB | GSM2203667 | M | White | 53 | PTB | Blood |
| PTB | GSM2203678 | F | ISC | 67 | PTB | Blood |
| PTB | GSM2203669 | F | White | 54 | PTB | Blood |
| PTB | GSM2203672 | M | Black | 43 | PTB | Blood |
| PTB | GSM2203658 | F | Black | 22 | PTB | Blood |
| PTB | GSM2203670 | M | ISC | 38 | PTB | Blood |
| PTB | GSM2203651 | M | ISC | 38 | PTB | Blood |
| PTB | GSM2203691 | M | White | 44 | PTB | Blood |
| PTB | GSM2203684 | F | ISC | 24 | PTB | Blood |
| PTB | GSM2203662 | M | ISC | 24 | PTB | Blood |
| PTB | GSM2203654 | F | ISC | 26 | PTB | Blood |
| PTB | GSM2203649 | M | Black | 42 | PTB | Blood |

| | | | | | | |
|---|---|---|---|---|---|---|
| PTB | GSM2203674 | F | White | 45 | PTB | Blood |
| PTB | GSM2203676 | M | White | 82 | PTB | Blood |
| PTB | GSM2203675 | M | ISC | 24 | PTB | Blood |
| PTB | GSM2203687 | M | ISC | 21 | PTB | Blood |
| PTB | GSM2203690 | M | ISC | 30 | PTB | Blood |
| PTB | GSM2203683 | M | Other | 26 | PTB | Blood |
| PTB | GSM2203686 | M | Black | 27 | PTB | Blood |
| EPTB | GSM2203563 | M | ISC | 33 | EPTB | Blood |
| EPTB | GSM2203550 | M | ISC | 33 | EPTB | Blood |
| EPTB | GSM2203567 | M | ISC | 29 | EPTB | Blood |
| EPTB | GSM2203547 | M | Black | 48 | EPTB | Blood |
| EPTB | GSM2203564 | M | ISC | 29 | EPTB | Blood |
| EPTB | GSM2203543 | M | ISC | 27 | EPTB | Blood |
| EPTB | GSM2203583 | M | Other | 50 | EPTB | Blood |
| EPTB | GSM2203548 | M | White | 25 | EPTB | Blood |
| EPTB | GSM2203584 | M | White | 30 | EPTB | Blood |
| EPTB | GSM2203585 | F | Other | 47 | EPTB | Blood |
| EPTB | GSM2203540 | M | ISC | 21 | EPTB | Blood |
| EPTB | GSM2203545 | M | ISC | 47 | EPTB | Blood |
| EPTB | GSM2203568 | M | ISC | 51 | EPTB | Blood |
| EPTB | GSM2203554 | M | ISC | 30 | EPTB | Blood |
| EPTB | GSM2203562 | F | Black | 23 | EPTB | Blood |
| EPTB | GSM2203542 | F | Black | 23 | EPTB | Blood |
| EPTB | GSM2203557 | M | White | 29 | EPTB | Blood |
| EPTB | GSM2203560 | F | ISC | 28 | EPTB | Blood |
| EPTB | GSM2203556 | M | ISC | 34 | EPTB | Blood |
| EPTB | GSM2203579 | F | Other | 41 | EPTB | Blood |
| EPTB | GSM2203553 | F | White | 20 | EPTB | Blood |
| EPTB | GSM2203576 | F | Black | 35 | EPTB | Blood |
| EPTB | GSM2203551 | M | ISC | 34 | EPTB | Blood |
| EPTB | GSM2203544 | F | ISC | 18 | EPTB | Blood |
| EPTB | GSM2203571 | F | White | 47 | EPTB | Blood |
| EPTB | GSM2203575 | F | Black | 51 | EPTB | Blood |
| EPTB | GSM2203573 | F | Other | 52 | EPTB | Blood |
| EPTB | GSM2203586 | M | ISC | 48 | EPTB | Blood |
| EPTB | GSM2203580 | F | White | 30 | EPTB | Blood |

| | | | | | | |
|---|---|---|---|---|---|---|
| EPTB | GSM2203549 | M | ISC | 34 | EPTB | Blood |
